# Transcriptomic responses in mouse brain exposed to chronic excess of the neurotransmitter glutamate

**DOI:** 10.1186/1471-2164-11-360

**Published:** 2010-06-07

**Authors:** Xinkun Wang, Xiaodong Bao, Ranu Pal, Abdulbaki Agbas, Elias K Michaelis

**Affiliations:** 1 Higuchi Biosciences Center, 2099 Constant Ave, The University of Kansas, Lawrence, KS 66047, USA; 2 Department of Pharmacology and Toxicology, 1251 Wescoe Dr., The University of Kansas, Lawrence, KS 66045, USA; 3 Current address: Department of Biochemistry, 1750 Independence Ave, Kansas City University of Medicine and Biosciences, Kansas City, MO 64106, USA

## Abstract

**Background:**

Increases during aging in extracellular levels of glutamate (Glu), the major excitatory neurotransmitter in the brain, may be linked to chronic neurodegenerative diseases. Little is known about the molecular responses of neurons to chronic, moderate increases in Glu levels. Genome-wide gene expression in brain hippocampus was examined in a unique transgenic (Tg) mouse model that exhibits moderate Glu hyperactivity throughout the lifespan, the neuronal *Glutamate dehydrogenase *(*Glud1*) mouse, and littermate 9 month-old wild type mice.

**Results:**

Integrated bioinformatic analyses on transcriptomic data were used to identify bio-functions, pathways and gene networks underlying neuronal responses to increased Glu synaptic release. Bio-functions and pathways up-regulated in Tg mice were those associated with oxidative stress, cell injury, inflammation, nervous system development, neuronal growth, and synaptic transmission. Increased gene expression in these functions and pathways indicated apparent compensatory responses offering protection against stress, promoting growth of neuronal processes (neurites) and re-establishment of synapses. The transcription of a key gene in the neurite growth network, the kinase *Ptk2b*, was significantly up-regulated in Tg mice as was the activated (phosphorylated) form of the protein. In addition to genes related to neurite growth and synaptic development, those associated with neuronal vesicle trafficking in the Huntington's disease signalling pathway, were also up-regulated.

**Conclusions:**

This is the first study attempting to define neuronal gene expression patterns in response to chronic, endogenous Glu hyperactivity at brain synapses. The patterns observed were characterized by a combination of responses to stress and stimulation of nerve growth, intracellular transport and recovery.

## Background

Glutamate (Glu) is the major excitatory neurotransmitter in the mammalian brain. Glu is released from nerve cell processes following electrical excitation, interacts with surface receptors in the synaptic region of another nerve cell, and initiates the influx of sodium and calcium ions across the membrane of the post-synaptic neuron. Such ion fluxes produce a change in the electrical properties of the post-synaptic nerve cell (sodium flux), as well as changes in intracellular signalling cascades that lead to altered gene expression (calcium entry) [1]. Thus, in addition to the rapid form of neuron-to-neuron communication through the actions of Glu as a transmitter, Glu also acts as a signalling molecule with long-lasting effects on neuronal structure and function. Glu neurotransmission is an important component of the modification of synaptic activity that is associated with the acquisition and storage of new information, i.e., learning and memory formation [2-4], and of the normal development of the nervous system [1,5,6].

Despite the multiple beneficial actions of Glu in the central nervous system (CNS) of mammals, it is also true that excessive excitation of neurons by Glu can lead to nerve cell damage and neurological dysfunction [7]. The consequences of hyper-excitation by Glu are swelling of the neuronal cell body and dendrites, destruction of organelles, and, ultimately, cell death [8]. It was proposed that pathological states in the CNS may result from either acute or chronic excessive release of Glu and over-activation of its post-synaptic receptors [9-13]. This suggestion is based, primarily, on the toxicity that follows acute exposure of nerve cells to excessive excitation by Glu. But, as we pointed out previously [14], acute treatments with Glu may not fully replicate the effects of chronic, localized, moderate excess release of Glu at synapses occurring throughout the lifespan of an organism.

To probe the effects of excess extracellular Glu concentrations in the brains of living organisms, three animal models were developed previously. The first two animal models are the null mutant mice for Glu transporters *Slc1a2 *(*Eaat2 *or *Glt-1*) and *Slc1a3 *(*Eaat1 *or *Glast*), and the third is a knockout for the tuberous sclerosis complex-1 gene (*Tsc1*) [15-18]. Both the *Slc1a2 *and *Tsc1 *null mutants have loss of function of Glu transporters and accumulate a large excess of Glu in the extracellular medium (1.5 to 32-fold normal levels) [16-18]. Since the Glu transporters are indispensable for normal brain development and function, knocking out two transporter genes in a mouse leads to excessive brain damage and embryonic lethality [15]. Knocking out either a single transporter gene, or the *Tsc1 *gene which affects the expression and function of Glu transporters, leads to dramatic reductions in the life span of the mutant mice (lifespan of 3-4 months) [16-18]. Based on these observations, it is clear that none of these mutant mice would be a good model for probing the effects of moderate, transient increases in extracellular Glu on neurons throughout the lifespan of an organism.

Recently, we have developed a transgenic (Tg) mouse model of chronic, moderate excess release of Glu in CNS synapses [14]. Using this model, we have shown that such release can decrease the number of synapses on nerve cell processes and suppress the electrical activation of such synapses in a physiological model of memory formation [14]. To achieve excess Glu formation in neurons and increased release from synaptic terminals in these animals, the gene for the enzyme glutamate dehydrogenase 1 (*Glud1*) was introduced under the control of the promoter for neuron-specific enolase (*Nse*), thus its over-expression occurs only in neurons [14]. Despite the increases in depolarization induced Glu release from nerve terminals, neuronal and synaptic losses were observed only in select populations of nerve cells. As similar changes in synaptic function and losses of select Glu-responsive neurons occur during normal brain aging and in neurodegenerative diseases, such as Alzheimer's disease [19-21], this line of Tg mice appeared to be a good model to study the transcriptomic effects of chronic Glu hyperactivity at synapses in order to begin to define the possible associated cell and molecular compensatory mechanisms to continuous, life-long, hyperactivity by the Glu-releasing neurons.

The neurons of the *Glud1 *Tg mice would be expected to make numerous transcriptomic changes in order to either adapt, compensate, or correct the disequilibrium of excess synaptic Glu stimulation during the lifetime of these animals. In addition to the increases in Glu release, the *Glud1 *mice may also be exposed to altered intracellular metabolic states as a result of the over-expression of the mitochondrial enzyme GLUD1. The genes whose expression would be significantly altered in *Glud1 *mice compared with age-matched wild type (wt) animals, and the molecular pathways and cellular functions that they represent, may help unravel the most substantial neuronal responses to the over-expression of *Glud1 *and the exposure of cells to excess Glu stimulation. In the present study, we present a detailed analysis of genome-wide expression differences between nine month-old *Glud1 *and wt mice. This analysis revealed mechanistic insights into the types of molecular functions, neurobiological pathways and gene-gene interactions that might influence the responses of CNS neurons to increased *Glud1 *mitochondrial activity and of neurons and glial cells to chronic, moderate excess of synaptic Glu release.

## Results and Discussion

### Changes in genomic transcription in hippocampal cells of *Glud1 *Tg mice

Comparative genome-wide transcriptional analyses of Tg and wt mice were performed on the hippocampus region of the brain of these animals. The rationale for the selection of this region for this study was two-fold: 1) the predominant neurotransmitter in the hippocampus is Glu; and 2) it is one of the brain regions in which clear structural and physiological changes were detected in the Tg mice [14]. The differences between *Glud1 *and wt mouse hippocampi included increases in synaptic Glu release, decreased long term potentiation of synaptic activity following high frequency stimulation of neurons, decreased number of dendritic processes (the receptive zones of neurons for incoming synaptic signals), decreased synaptic sites on dendrite protrusions (the dendrite spines), and decreased number of nerve terminals that form such synapses. Furthermore, the hippocampal region is particularly vulnerable to aging and neurodegenerative diseases, and this vulnerability may result from chronic Glu hyperactivity in this brain region [21].

Nine mo-old *Glud1 *Tg mice were used in the study of whole genome gene expression. When compared to wt mice of the same age, *Glud1 *mice at this age exhibit some initial signs of neuronal loss and damage to their dendrites and nerve terminals, but not as extensive as those observed at older ages (e.g., 20 mo-old) [14]. Thus, at the age of nine months it would be possible to probe for changes in gene expression that may represent adaptive, compensatory or corrective responses of hippocampus cells to chronic Glu hyperactivity, while at the same time minimizing the impact of neuronal death on the results of gene array analyses.

A distinct transcriptional profile was found in *Glud1 *Tg mice as compared with that of wt animals. Analysis of the microarray data using condition tree revealed two distinct clusters, one corresponding to the group of *Glud1 *mice and the other to that of wt mice. This observation supported the idea that neurons and glia in the brains of the *Glud1 *Tg mice were responding to the chronic over-expression of *Glud1 *in neurons by exhibiting a different pattern of DNA transcription than that seen in wt mice.

**Figure 1:**
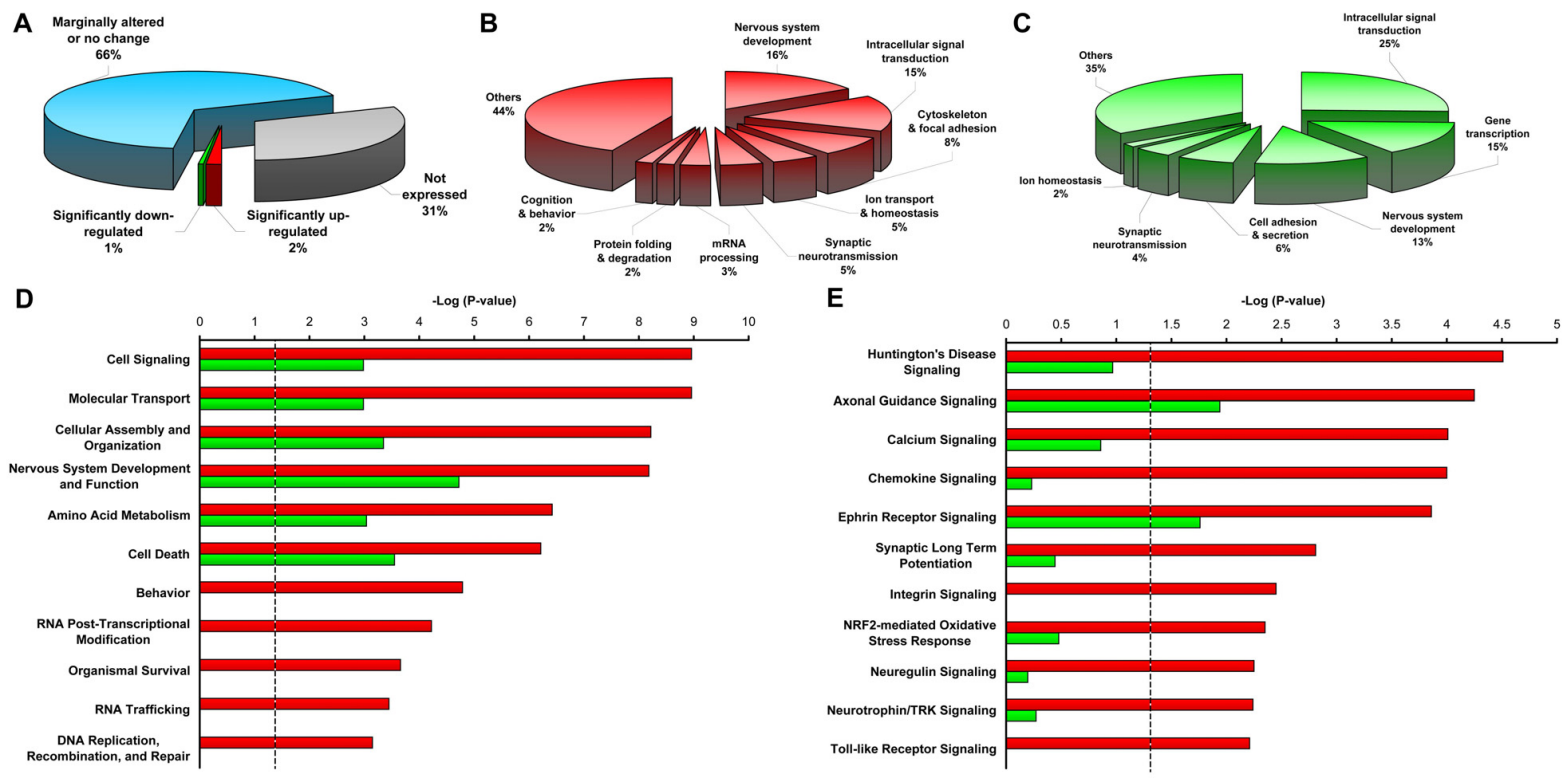
**Enrichment analysis of functional groups of up- and down-regulated genes associated with chronic glutamate hyperactivity**. (A) Approximately 2% of the over 39,000 gene transcripts surveyed by Affymetrix Mouse Genome 430 2.0 Array were identified to be significantly up-regulated, and 1% to be down-regulated, with an FDR of 0.9%. (B, C) Gene Ontology (GO) analysis of up- (B) and down-regulated (C) biological processes associated with chronic glutamate hyperactivity. This analysis was conducted with GO-elite using hypergeometric distribution. A Z score > 1.96 or < -1.96 and a permute P value < 0.05, were considered to be statistically significant. (D, E) Comparative analyses of over-represented bio-functions and canonical pathways, respectively, in up- and down-regulated genes using Ingenuity Pathway Analysis (IPA). Significance calculated by IPA was plotted as -log *P *value and the dashed line corresponds to the threshold *P *= 0.05.

Using the Affymetrix GeneChip Mouse Genome 430 2.0 array that contains 45,037 probesets of over 39,000 transcripts, a total of 1,018 probesets were identified as being significantly altered in *Glud1 *Tg mice. Among these differentially expressed probesets, 707 were more highly expressed in Tg than wt mice (up-regulated), and 311 showed the opposite pattern (down-regulated) (Figure 1A). Some of the prominent up- or down-regulated genes in *Glud1 *Tg mice are shown in Additional file 1, with the complete list of differentially expressed probesets available in Additional file 2. Of the 1,018 differentially expressed probesets, only 9 were expected to be false positives, i.e., the false discovery rate (FDR) was 0.9%.

### Transcriptomic changes in neurobiological processes, functions, and pathways in response *to Glud1 *over-expression

To uncover neurobiological and cellular processes that were significantly altered in *Glud1 *mouse hippocampus, we first carried out Gene Ontology (GO) analyses on the up- or down-regulated genes identified above. The GO biological processes that were significantly enriched in up-regulated genes in *Glud1 *mice compared with wt mice are shown in Figure 1B, while those that were enriched in down-regulated genes are shown in Figure 1C. The specific genes within the GO categories shown in Figure 1B and 1C, are listed in Table 1. As shown in Figure 1B and 1C, as well as in Table 1, there were eight categories of biological processes that were significantly enriched in up-regulated genes and six GO categories significantly enriched in down-regulated genes in *Glud1 *mice. Among these categories, four (*Nervous system development, Intracellular signal transduction, Synaptic neurotransmission*, and *Ion homeostasis*) were enriched in both up- and down-regulated genes (Figure 1B, C; Table 1), with the up-regulated genes in *Glud1 *mice outnumbering those that were down-regulated in the respective categories. Four of the other GO categories were enriched only in up-regulated genes in Tg mice. These four categories were: *mRNA processing*, *Protein mis-folding correction and degradation*, *Cytoskeleton and focal adhesion*, and *Cognition and behavior *(Table 1).

**Table 1:** Genes associated with the Gene Ontology (GO) biological processes shown in Figure 1B and 1C

GO Biological Processes	Associated Genes
***A) Up-regulated GO biological processes***	

**Nervous system development (70 genes)**	*Actr3, Acvr1, Ank3, Apc, Aplp2, Arc, Arhgef2, Arpc2, Atp2b2, Atrx, B3gnt2, Bcl11b, Brsk2, Btg2, Cacna1c, Centb2, Cttn, Diap1, Dpysl2, Egr2, Elavl3, Enah, Epha3, Epha4, Epha6, Epha7, Fos, Fut8, Gja1, Grin2a, Hipk2, Itga8, Kif5c, Klhl24, Lefty1, Mbp, Mef2a, Mib1, Ndel1, Ndst1, Nfib, Nisch, Npnt, Nrcam, Nrp1, Nrp2, Nrxn3, Ntrk2, Ntrk3, Pafah1b1, Pbx1, Pcsk2, Pex2, Prkca, Prkg1, Ptk2, Rsc1a1, Rtn4, Scn2a1, Scn8a, Sema5a, Sh3rf1, Slit3, Tacc1, Top2b, Trpm7, Wasl, Yy1, Zbtb16, Zeb2*
**Intracellular signal transduction (68)**	*Aak1, Abl2, Acvr1, Add1, Add2, Akt3, Aplp2, Arhgef12, Arhgef2, Atp2b2, Brap, Brsk2, Camk2a, Camk2b, Centb2, Centg2, Csnk1a1, Diap1, Dock10, Dock9, Dyrk1a, Epha3, Epha4, Epha6, Epha7, Fert2, Frap1, Fut8, Hipk2, Hrasls, Itga8, Kalrn, Ksr1, Map2k4, Mapk14, Mapk8, Mtmr1, Mtmr3, Mtmr9, Myo9b, Ndst1, Net1, Nisch, Npnt, Nrp1, Ntrk2, Ntrk3, Pafah1b1, Pftk1, Phip, Phka2, Ppp3ca, Prkca, Prkg1, Ptk2, Ptk2b, Ptprj, Ptprs, Rabgap1, Rps6kb1, Rutbc3, Slc8a2, Slk, Srpk2, Tbc1d15, Tlk2, Trpm7, Uhmk1*
**Cytoskeleton & focal adhesion (38)**	*Abl2, Actr3, Add1, Add2, Apc, Arc, Arhgef2, Arpc2, Cap2, Capn3, Centb2, Clasp1, Clip1, Coro1c, Diap1, Dmd, Dst, Enah, Enc1, Epb4.1l1, Frmd4b, Gmfb, label, Mtap7, Myo9b, Ndel1, Nisch, Pafah1b1, Polb, Ptk2, Ptk2b, Spire1, Tacc1, Tpm1, Trpm7, Wasf3, Wasl, Wdr1*
**Ion transport & homeostasis (23)**	*Atp1a1, Atp2b2, Atp2c1, Atp6v0d1, Atp8a1, Atp9b, Cacna1c, Cacna1d, Cacna1h, Cacna2d1, Cacnb2, Camk2a, Camk2b, Grin2a, Grin2b, Ppp3ca, Ryr1, Scn2a1, Scn3b, Scn8a, Slc8a2, Trpc4, Trpm7*
**Synaptic neurotransmission (21)**	*Arc, Atp2b2, Atxn1, Camk2a, Dlg1, Dlg2, Dlg4, Erc2, Exoc4, Exoc5, Gabbr1, Gabrb2, Gabrb3, Grin2a, Grin2b, Magi2, Nrxn3, Ppp3ca, Rps6kb1, Snca, Vps33a*
**mRNA processing (15)**	*Adar, Cdc40, Cpsf6, Cugbp2, Lsm11, Prpf39, Prpf40a, Sfpq, Sfrs10, Sfrs12, Sfrs8, Srpk2, Srrm2, Syncrip, Tra2a*
**Protein mis-folding correction & degradation (9)**	*Ahsa1, Ahsa2, Dnajc1, Usp12, Usp19, Usp20, Usp32, Usp47, Usp7*
**Cognition & behavior (8)**	*Atxn1, Cacna1c, Epha4, Grin2a, Grin2b, Itga8, Pafah1b1, Scn8a*

***B) Down-regulated GO biological processes***	

**Intracellular signal transduction (57)**	*AW551984, Acvr2b, Adra2a, Angpt1, Anxa1, Arhgap6, Bmper, Cacnb4, Calca, Car8, Cckbr, Cd24a, Cdc42ep1, Cit, D0H4S114, Dcx, Ddit4l, Ddr2, Depdc6, Gnb4, Gng4, Gpr101, Gpr103, Gpr83, Gulp1, Hrh1, Igf1, Khdrbs1, Nr2f2, Pde1c, Pde9a, Pgr, Plcxd3, Plek, Ppard, Ptprd, Rab3b, Reln, Rfxank, Rgs17, Rgs2, Rora, Rspo2, Sh2d3c, Shd, Slc9a3r2, Sparc, Stmn1, Tacr3, Tbc1d8, Tgm2, Timp2, Trhr, Unc13c, Unc5d, Wnt4, Wnt7a*
**Gene transcription (33)**	* 6430502M16Rik, Ankrd56, Arx, Btbd11, Cutl1, Cutl2, Etv6, Foxp2, Hes5, Hivep1, Hoxc6, Ikzf4, Irf6, Khdrbs1, Klf5, Lbh, Ldb2, Lhx2, Maf, Mef2c, Mrg1, Nr2f2, Pbx3, Pgr, Ppard, Prrx1, Rora, Satb1, Satb2, Ssbp2, Trim66, Tshz2, Tshz3*
**Nervous system development (29)**	*Arx, Atp1a2, Calca, Car10, Cd24a, Cd44, Cit, Cobl, Cutl1, Dcx, Efna5, Foxp2, Gap43, Grin3a, Hes5, Igf1, Lhx2, Nr2f2, Nts, Pex13, Pgr, Ppard, Reln, Rora, Stmn1, Timp2, Tnnt2, Wnt4, Wnt7a*
**Cell adhesion & secretion (14)**	*Amn, Anxa1, Cacnb4, Calca, Cd24a, Cplx3, Cutl1, Exoc6, Ppard, Rab3b, Rims3, Tgm2, Unc13c, Wnt7a*
**Synaptic neurotransmission (10)**	*Atp1a2, Cacnb4, Cckbr, Cplx3, Gpr103, Gpr83, Rims3, Slc17a6, Tacr3, Wnt7a*
**Ion homeostasis (4)**	*Atp1a2, Cacnb4, Calca, Cd24a*

Comparative bio-function analysis between Tg and wt mouse hippocampus genes was also performed using the Ingenuity Pathway Analysis (IPA) software. Major bio-functions significantly enriched in up- or down-regulated genes in Tg mice are shown in Figure 1D and are listed in Additional file 3. Of the eleven bio-functions, six were enriched in both up- and down-regulated genes in Tg mice, and five were enriched only in up-regulated genes in the *Glud1 *mice. In addition, the statistical significance values (shown as -Log *P*) for up-regulated genes were significantly greater than those for the down-regulated genes in bio-functions populated by both up- and down-regulated genes in *Glud1 *mice (Figure 1D).

As indicated in Figure 1D, the bio-functions identified by the IPA analysis were similar to the biological processes identified by the GO analysis, including *Cell signalling*, *Nervous system development*, *RNA post-transcriptional modification and RNA trafficking*, *Behavior*, *Cellular assembly and organization*, and *Molecular transport*. The genes in these bio-functions (Additional file 3) were, for the most part, the same genes identified by the GO analysis (Table 1). Bio-functions identified by the IPA analysis but not by the GO analysis included those of *Amino acid metabolism*, *Cell death*, *Organismal survival*, and *DNA replication, recombination, and repair *(Figure 1D and Table 2).

**Table 2:** Genes associated with IPA bio-functions and canonical pathways that are different from the GO categories shown in Table 1

Bio-Functions	Associated Genes
**Molecular Transport**	
Up-regulated genes (41)	*Anp32a, Atp1a1, Atp2b2, Atp2c1, Atxn1, Cacna1c, Cacna1d, Cacna1h, Cacna2d1, Cacnb2, Camk2a, Camk2b, Erc2, Frap1, Gja1, Gnaq, Gpam, Grin2a, Grin2b, Htr1a, Kcna6, Kcnd2, Kcnip2, Kcnq1, Kcnq2, Lyk5, Nrxn3, Ntrk2, Ppp3ca, Ptk2, Rgs14, Ryr1, Scd, Scn2a, Scn8a, Smg6, Snca, Trpc4, Trpm7, Uhmk1, Ywhae*
Down-regulated genes (18)	* Adra2a, Atp1a2, Calca, Camk2d, Cast, Cav2, Cckbr, Dcn, Gap43, Igf1, Igfbp3, Nts, Rgs2, Satb1, Slc17a6, Trhr, Vip, Wnt7a*
**Cellular Assembly and Organization**	
Up-regulated genes (100)	*Actb, Adam17, Add1, Ank3, Anp32a, Ap2a2, Apc, Arf3, Arhgef12, Arhgef2, Arpc2, Atp2b2, Atp2c1, Bcl11b, Bin1, Cacna1c, Cacnb2, Camk2a, Cap2, Capn10, Centb2, Centg2, Clasp1, Clip1, Coro1c, Cttn, Diaph1, Dlg1, Dlg4, Dmd, Dnaja3, Dpysl2, Dsp, Dst, Eif4a1, Eif4e, Enah, Enc1, Epha4, Erc2, Exoc5, Frap1, Gas7, Gja1, Gnaq, Gng2, Gosr1, Gpm6a, Grin2a, Grip1, Htr1a, Itga8, Junb, Kalrn, Kcnq1, Klf2, Kpnb1, Ksr1, Map2, Map7, Mapk14, Mapk8, Mbp, Myo9b, Napg, Nav1, Ncam1, Ndel1, Neo1, Net1, Nfia, Nisch, Nmt1, Nrcam, Nrp1, Ntrk2, Ntrk3, Pafah1b1, Pex5l, Picalm, Prkg1, Psap, Ptk2, Ptk2b, Reps1, Rictor, Rims1, Rps6kb1, Rtn4, Sec24b, Sema5a, Slit3, Snca, Spire1, Top2b, Tpm1, Trpc4, Wasf3, Wasl, Ywhaz*
Down-regulated genes (26)	*Cacnb4, Calca, Cast, Cav2, Cd24, Cd44, Cdc42ep1, Cdh13, Cit, Cntn4, Ctgf, Dcx, Efna5, Gap43, Grin3a, Igf1, Igfbp3, Klf5, Nts, Ppard, Reln, Rgs2, Sdc2, Sparc, Stmn1, Wnt7a*
**Amino Acid Metabolism**	
Up-regulated genes (35)	*Acvr1, Acvr2a, Akt3, B3gnt2, Brsk2, Btg2, Camk2a, Camk2b, Col4a3bp, Csnk1a1, Dusp6, Dyrk1a, Fer, Frap1, Gmfb, Hipk2, Kalrn, Large, Lyk5, Mapk14, Mapk8, Mtmr3, Ndst1, Phka2, Ppp3ca, Prkca, Prkg1, Ptk2b, Ptprd, Ptprj, Rps6kb1, Srpk2, St6galnac3, Tlk2, Trpm7*
Down-regulated genes (6)	* Ass1, Igf1, Igfbp3, Nts, Reln, Slc17a6*
**Cell Death**	
Up-regulated genes (99)	*Acvr1, Akt3, Aldh2, Ap2a2, Apc, Arc, Atf2, Atf6, Atp1a1, Atp2c1, Atrx, Atxn1, Bclaf1, Bin1, Btg2, Cacna1c, Cacnb2, Calb1, Camk2a, Capn10, Chka, Csnk1a1, Dmd, Dnaja3, Dsp, Dusp6, Egr1, Egr2, Eif4e, Fem1b, Fer, Fos, Frap1, Fubp1, Fus, Gabbr1, Gja1, Gmfb, Gnaq, Gng2, Grin2a, Hdac2, Hipk2, Hpca, Junb, Klf2, Ksr1, Lrig1, Map2k4, Mapk14, Mapk8, Mbp, Mef2a, Mgat3, Ncam1, Ndel1, Ndst1, Nptx1, Nr4a1, Nrcam, Nrf1, Nrp1, Ntrk2, Ntrk3, Pafah1b1, Phip, Polb, Ppp1r13b, Ppp3ca, Prkca, Psap, Ptk2, Ptk2b, Rad23b, Rasgrp1, Rbm5, Rps6kb1, Rraga, Rtn4, Scn2a, Scn3b, Sh3rf1, Ski, Slk, Snca, Tacc1, Tfrc, Tnfrsf25, Tnks2, Tpm1, Ube2k, Ubtf, Usp7, Vps33a, Ywhae, Yy1, Zbtb16, Zeb2, Zfr*
Down-regulated genes (37)	* Acvr2b, Angpt1, Anxa1, Atp1a2, Calca, Camk2d, Casp1, Cast, Cd24, Cd44, Cit, Ctgf, Dcn, Ecop, Etv6, Gulp1, Hoxc6, Igf1, Igfbp3, Igfbp6, Klf5, Mef2c, Nedd9, Phlda1, Plagl1, Ppard, S100a10, Satb1, Sdc2, Sparc, Stmn1, Tgm2, Timp2, Tmsb10, Vip, Wnt7a, Wwox*
**Organismal Survival**	
Up-regulated genes (44)	*Adar, Akt3, Apc, Aplp2, Cacnb2, Dmd, Dnaja3, Dsp, Dyrk1a, Egr2, Enah, Fus, Fut8, Gabrb3, Gja1, Gnaq, Grip1, Hapln1, Hipk2, Klf2, Magi2, Map2k4, Mapk14, Mapk8, Mef2a, Nfia, Nrf1, Nrp1, Ntrk2, Ntrk3, Pbx1, Pnpla6, Polb, Prkca, Ptk2, Ptprs, Rad23b, Ski, Spred2, Top2b, Wasl, Wdr1, Yy1, Zfr*
**RNA Trafficking**	
Up-regulated genes (3)	*Eif3a, Eif4a1, Eif4e*
**DNA Replication, Recombination, and Repair**	
Up-regulated genes (13)	*Atf2, Atp1a1, Fos, Gnaq, Gng2, Iqgap2, Kpnb1, Mapk8, Myo9b, Ptk2, Ptk2b, Ubtf, Yy1*

**Canonical Pathways**	**Associated Genes**

** Huntington's Disease Signalling**	
Up-regulated genes (Total number: 17)	*Akt3, Ap2a2, Atf2, Capn10, Capn3, Dlg4, Frap1, Gnaq, Gng2, Gosr1, Grin2b, Hdac2, Map2k4, Mapk8, Napg, Prkca, Snca*
Down-regulated genes (5)	*Casp1, Gng4, Igf1, Pik3r5, Tgm2*
**Axonal Guidance Signalling**	
Up-regulated genes (23)	*Adam17, Akt3, Arhgef12, Arpc2, Dpysl2, Eif4e, Epha3, Epha4, Epha6, Epha7, Gnaq, Gng2, Kalrn, Nrp1, Ntrk2, Ntrk3, Ppp3ca, Prkca, Ptk2, Rtn4, Sema5a, Slit3, Wasl*
Down-regulated genes (10)	*Arhgef15, Efna5, Gng4, Igf1, Pik3r5, Sdc2, Sema4a, Unc5d, Wnt4, Wnt7a*
**Chemokine Signalling**	
Up-regulated genes (9)	*Camk2a, Camk2b, Fos, Gnaq, Mapk14, Mapk8, Prkca, Ptk2, Ptk2b*
Down-regulated genes (1)	*Camk2d*
**Ephrin Receptor Signalling**	
Up-regulated genes (14)	*Akt3, Arpc2, Atf2, Epha3, Epha4, Epha6, Epha7, Gnaq, Gng2, Grin2a, Grin2b, Kalrn, Ptk2, Wasl*
Down-regulated genes (6)	*Angpt1, Arhgef15, Efna5, Gng4, Sdc2, Sh2d3c*
** Synaptic Long Term Potentiation**	
Up-regulated genes (9)	*Atf2, Cacna1c, Camk2a, Camk2b, Gnaq, Grin2a, Grin2b, Ppp3ca, Prkca*
Down-regulated genes (2)	*Camk2d, Grin3a*
**Integrin Signalling**	
Up-regulated genes (12)	*Actb, Akt3, Arf3, Arpc2, Capn10, Capn3, Itga8, Map2k4, Mapk8, Ptk2, Tspan5, Wasl*
**NRF2-mediated Oxidative Stress Response**	
Up-regulated genes (11)	*Actb, Dnaja3, Dnajb5, Dnajc1, Fos, Junb, Map2k4, Mapk14, Mapk8, Prkca, Ube2k*
Down-regulated genes (3)	*Maf, Pik3r5, Slc35a2*
** Neuregulin Signalling**	
Up-regulated genes (7)	*Adam17, Akt3, Dlg4, Frap1, Nrg3, Prkca, Rps6kb1*
Down-regulated genes (1)	*Dcn*
**Neurotrophin/TRK Signalling**	
Up-regulated genes (6)	*Atf2, Fos, Map2k4, Mapk8, Ntrk2, Ntrk3*
Down-regulated genes (1)	*Pik3r5*
**Toll-like Receptor Signalling**	
Up-regulated genes (5)	*Fos, Map2k4, Mapk14, Mapk8, Tollip*

IPA analysis was also used to determine canonical biological pathways that were significantly enriched with either up- or down-regulated genes in *Glud1 *compared with wt hippocampus cells. Eleven pathways were identified (Figure 1E and Additional file 4). Use of the IPA canonical pathway analysis led to the identification of some biological pathways that did not relate to either the categories of biological processes identified by means of the GO analysis or to the bio-functions identified by IPA. Yet, some of these pathways were enriched with genes that were components of either GO categories or IPA-identified bio-functions, such as *Axonal guidance signalling*, *Calcium signalling*, *Ephrin receptor*, *Synaptic long term potentiation*, *Integrin signalling*, and *Neurotrophin/TRK signalling *(Additional file 4). But, several of the pathways identified by IPA analysis provided new information into the effects of chronic *Glud1 *over-expression and mild Glu hyperactivity on hippocampal neurons that was not uncovered by the GO or IPA bio-function analyses (Table 2). These pathways were *Huntington's disease signalling*, *Chemokine signalling*, *NRF2-mediated oxidative stress response*, *Neuregulin signalling*, and *Toll-like receptor signalling *(Figure 1E and Table 2).

Overall, the GO and IPA analyses identified some key biological and neurobiological processes, functions and pathways that appeared to be altered in *Glud1 *mice primarily through gene up-regulation. Looking at these categories in their totality, it would appear that hippocampal cells in the Tg mice were under some level of stress to which they responded by activating intracellular signals (*Intracellular signal transduction *and *Cell signalling*), signals that may lead to cell protection from damage or death (*Protein mis-folding correction & degradation *and *Cell death*), signals that are associated with inflammatory responses and cell survival (*Chemokine signalling *and *Toll-like receptor signalling*), and signals that mediate responses to oxidative stress (*NRF2-mediated oxidative stress response*). In addition to the signalling pathways enumerated above, there was also a signalling pathway related to a severe neurological disorder, the *Huntington's disease signalling *pathway.

#### Huntington's disease signalling pathway

This pathway was most significantly enriched with up-regulated genes in the *Glud1 *mice (Figure 1E). Huntington's disease is an autosomal dominant hereditary neurological disorder caused by an expansion of triplet repeats in the gene for the protein Huntingtin. Huntington's disease does not usually affect hippocampal neurons, therefore, the enhanced expression of genes in this pathway in *Glud1 *mice may be a reflection of molecular processes that are potentially related to the cause or the progression of the disease. A current view of the molecular and cellular defects associated with this disease is that the abnormal Huntingtin protein leads to changes in vesicle trafficking in neurons [22]. Among the genes up-regulated in the *Glud1 *mice was *Snca *(Alpha-Synuclein), a protein involved in neurotransmitter (dopamine) release from nerve terminals and in the integration of signal transduction and membrane vesicle trafficking at nerve terminals. Some of the other up-regulated genes in the *Huntington's disease signalling *pathway included the gene *Ap2a2 *(Adaptor Protein Complex AP-2, Alpha 2 Subunit; also known as Huntingtin-Interacting Protein J). The AP2A2 protein, together with Huntingtin, interacts with the protein Clathrin in the process of endocytosis of membrane vesicles [23]. Another significantly up-regulated gene in the same pathway, *Gosr1*, encodes a member protein of the soluble *N*-Ethylmaleimide-Sensitive Factor Attachment Protein Receptor (SNARE) complex that links endocytic vesicles to the *trans*-Golgi network in cells [24]. Finally, *Napg *(*N*-Ethylmaleimide Sensitive Fusion Protein Attachment Protein Gamma), important in vesicle trafficking in cells [25], was also an up-regulated gene in Tg mice. Thus, it appeared that hippocampal neurons responded to excess expression of *Glud1 *and activation of Glu neurotransmission by up-regulating several genes related to vesicular trafficking, genes that are also part of the Huntington's disease pathway.

#### Intracellular signal transduction

Examples of genes involved in intracellular signal transduction related to stress and to either apoptosis or cell survival included the genes for two stress kinases, c-Jun N-terminal Kinase (*Mapk8*) and p38 MAP Kinase (*Mapk14*), the gene for an anti-apoptotic protein that protects neurons from Glu-induced apoptosis, Myocyte Enhancer Factor 2a (*Mef2a*) [26], the gene for a member of the mitochondrial heat shock factor 40 protein family (*Dnaja3*, DnaJ [Hsp40] Homolog, Subfamily A, Member 3) with both pro-apoptotic and cell survival-enhancing functions [27], and the gene for a protein that affects brain development and neuronal survival and differentiation, the Dual Specificity Tyrosine-Phosphorylation Regulated Kinase 1a (*Dyrk1a*) [28]. As indicated, some of the genes have dual or multiple functions, for example, *Dnaja3 *may function as a pro-apoptotic or as a survival-enhancing protein, and it may also function as a nerve cell process (neurite) growth-promoting gene [27]. The same is true for *Dyrk1a *whose absence stunts brain growth, development and neurite elongation, yet its presence in excess, as it occurs in trisomy 21 (Down's syndrome), may retard neuronal growth and differentiation [28,29]. We assume that in *Glud1 *mice, the primary role of these genes is in enhancing neuronal growth and survival.

#### Stress response

Congruent with the overall up-regulation of stress response, the *NRF2-mediated oxidative stress response *pathway was also enriched with genes whose expression was up-regulated. In addition to the *Dnaja3 *for Heat Shock Protein 40, a related heat shock protein gene, *Dnajb5 *(DnaJ [Hsp40] Homolog, Subfamily B, Member 5) was also significantly up-regulated in Tg compared with wt mice (Table 2). Represented also in this pathway were the genes for the transcription factors *Fos *and *JunB*, which are known to be up-regulated following synaptic activation of nerve cells, during learning of a new task, and also under conditions of stress and neuronal damage [30,31]. Increased expression of these factors may be sustained for a long period of time following neuronal injury [32]. Thus, their up-regulation in Tg mice may be considered as a trace of neuronal injury in the hippocampus, possibly, as a result of chronic excess synaptic Glu release in the Tg mice.

#### Protein mis-folding correction and degradation

Enhanced expression of genes for heat shock proteins described above is not only indicative of a stress response but also of an overall increase in protein refolding or targeting for degradation. The category of *Protein mis-folding correction & degradation *(Table 2) included three chaperone proteins distinct from the two heat shock proteins (*Dnaja3 *and *Dnajb5*) described above. They were *Ahsa1 *(Activator of Heat Shock 90 kDa Protein ATPase Homolog 1), *Ahsa2*, and *Dnajc1 *(DnaJ [Hsp40] Homolog, Subfamily C, Member 1). Heat Shock Protein 90 is emerging as a very important chaperone in nerve cells and its activity may determine the viability of neurons in neurodegenerative diseases, such as Alzheimer's disease [33]. The Activators of Heat Shock 90 kDa Proteins (AHSA1 and AHSA2) are important regulators of the activity of this class of chaperones [34] and may be indicators of cellular response to excessive protein damage.

In addition to the over-expression of chaperone activator and chaperone protein genes in the Tg mice, six genes involved in protein degradation were also part of the GO category of *Protein mis-folding correction & degradation*. These are the genes for Ubiquitin-Specific Peptidases, including USP7, USP12, USP19, USP20, USP32 and USP47, which degrade proteins following the attachment of ubiquitin molecules to the proteins. In addition to these Ubiquitin-Specific Peptidase genes, the genes for Ubiquitin-Conjugating Enzymes, UBE2K and UBE3A, which covalently attach ubiquitin molecules to proteins destined for degradation, were also up-regulated in Tg mice. The up-regulation of genes related to the correction of mis-folded proteins (i.e., chaperone activity) and those involved in the degradation of mis-folded proteins (i.e., ubiquitination and proteolysis), was indicative of a heightened level of protein modification, defects in folding, and likely loss of function of proteins in the *Glud1 *hippocampus.

The transcriptomic data reported here on the up-regulation of protein ubiquitination genes matched our previous observations of large accumulations of ubiquitinated proteins in neurons of the same region in older *Glud1 *mice [14]. It should be noted that in this study, the transcriptional up-regulation of protein ubiquitination genes occurred by nine months of age, that is, it preceded the accumulation and aggregation of ubiquitinated proteins (not observed until the Tg mice were at sixteen months of age [14]).

#### Chemokine and Toll-like receptor signalling

The significant up-regulation of two signalling pathways, *Chemokine signalling *and *Toll-like receptor signalling*, also reveals increased cell responses to stress in the Tg mice. Chemokines (short form of chemo-attractant cytokines) are peptides that control inflammatory responses in the immune system. In the brain, chemokines also activate pathways that lead to cell survival, cell adhesion, cell polarity, and synaptic transmission, that is, they control many of the GO biological processes or IPA bio-functions identified above (Tables 1 and 2) [35]. The genes included in the *Chemokine signalling *pathway (Table 2) were those of post-receptor signalling and included genes for the focal adhesion kinases *Ptk2b *(Protein Tyrosine Kinase 2 Beta) and *Ptk2 *(Protein Tyrosine Kinase 2), calcium-diacylglycerol-activated protein kinase C alpha (*Prkca*), stress kinases *Mapk8 *and *Mapk14*, and the calcium/calmodulin-dependent protein kinases *Camk2a *and *Camk2b*. Up-regulation of these chemokine signalling pathway genes would be expected to lead to activation of cell survival, growth, and synaptic transmission in neurons [35]. Furthermore, some of the same proteins are also involved in the Toll-like receptor signalling pathway [36], especially the stress-activated kinases *Mapk8 *and *Mapk14*.

Toll-like receptors are normally activated by macromolecules associated with bacteria or viral particles and their activation leads to the innate immune responses in the immune system. In the brain, Toll-like receptors may be activated by macromolecules released from damaged cells [36] and they may serve an important function in clearing the extracellular space of potentially damaging molecules, such as the amyloid beta that accumulates in Alzheimer's disease [37]. Thus, stimulation of the chemokine and Toll-like receptor signalling pathways in brain cells may result from activation of stress signals within neurons and from breakdown products released following injury to neurons and glial cells. Overall, the transcriptomic profile of the *Glud1 *Tg hippocampal cells is a pattern of mounting defenses against cellular stress, enhancing cell survival/growth/differentiation and, as will be described below, preservation or re-establishing normal neuronal function, especially synaptic transmission.

#### Amino acid metabolism functions

Noticeable was the absence of either up-regulated or down-regulated genes that might be directly involved in Glu-glutamine metabolism or mitochondrial metabolic activity in *Glud1 *mice. The biological function amino acid metabolism (Table 2) had several up-regulated genes but these genes represented various types of kinases and phosphatases, as well as proteins involved in cell growth, differentiation, proliferation, and glycoside synthesis. Only one gene directly related to amino acid metabolism, *Ass1 *(Arginino-Succinate Synthetase 1), was down-regulated (Table 2). This enzyme is part of the urea cycle, a metabolic pathway that is also linked to Glu metabolism and to GLUD1 activity in mitochondria [38]. Down-regulation of this enzyme may represent neuronal compensatory responses that would lead to reduced 2-oxoglutarate and glutamate formation.

It is also particularly significant that one of the most down-regulated genes in *Glud1 *mice was the gene *Slc17a6 *(Solute Carrier Family 17, Member 6; or Vesicular Glutamate Transporter) (Table 2). This is the transporter for Glu uptake and storage in synaptic vesicles of neurons. The decreases in the vesicular Glu transporter gene expression may represent another compensatory response in neurons aimed at decreasing the amount of Glu released at the synapse.

### Transcriptomic changes on synapse formation, synaptic transmission, and neurite outgrowth and elongation

Neuronal growth, differentiation and preservation of synaptic transmission were also reflected in the GO categories, IPA bio-functions and canonical pathways that were enriched with genes differentially expressed in Tg mice. The genes in these categories were mostly up-regulated in the Tg mouse hippocampus and fell into one of the following biological processes, bio-functions or pathways: *Nervous system development*, *Synaptic neurotransmission*, *Cytoskeleton and focal adhesion*, *Axonal guidance signalling, Ephrin receptor signalling*, *Integrin signalling*, *Neuregulin signalling*, *Neurotrophin/TRK signalling*, and *Synaptic long-term potentiation*. All of these categories are related to growth of nerve processes, differentiation of neurons, synapse formation, and synaptic transmission. *Synaptic neurotransmission*, one of the significantly altered GO biological processes in *Glud1 *mice (Table 1), contained two of the most up-regulated genes in *Glud1 *mice, those for the two Glu-activated synaptic receptors, the *N*-methyl-d-aspartate (NMDA) Receptor Subunits 2A and 2B (*Grin2A *and *Grin2B*). The IPA pathway analysis also identified *Synaptic long term potentiation*, a pathway related to activation of synaptic transmission, as one of the pathways that were significantly enriched with up-regulated genes (including *Grin2a *and *Grin2b*) (Table 2). The increased transcription for the two NMDA receptor proteins might appear to be counterintuitive in neural cells that are suffering from excess synaptic release of Glu, because NMDA receptors are highly permeable to Ca^2+ ^and the excess Ca^2+ ^entry is known to trigger oxidative stress and neuronal death [9]. It is quite likely, however, that the increased expression of NMDA receptor genes, especially *Grin2b*, was a reflection of the growth of new synapses [39]. The observed increases in gene expression for the synaptic membrane scaffold proteins (*Dlg1, Dlg2*, and *Dlg4*), and a protein involved in synapse strengthening (*Arc*, Activity-Regulated Cytoskeleton-Associated Protein), also fit a pattern of new synapse growth.

It was noted that gene expression for one type of NMDA receptor proteins, that of *Grin3a*, was decreased (down-regulated genes, *Nervous system development*, Table 1). Because GRIN3A has a negative effect on synapse formation [40], the decreases in *Grin3a *transcription would fit with the hypothesis of neuronal compensatory responses in *Glud1 *Tg mice that lead to increases in new synapse formation (i.e., through decreases in a negative regulator of synapse formation). The genes for several other Glu receptors were marginally altered, with some down-regulated (*Grm4 *[Glu Receptor, Metabotropic 4]: 1.27-fold) and *Grm8 *[Glu Receptor, Metabotropic 8]: 1.37-fold), while others were up-regulated (*Gria1 *[Glu Receptor, Ionotropic, AMPA 1]: 1.25-fold, *Gria3 *[Glu Receptor, Ionotropic, AMPA 3]: 1.27-fold, and *Grik2 *[Glu Receptor, Ionotropic, Kainate 2]: 1.27-fold). The Glu receptor family contains many members with divergent functions, differing kinetics of activation, differing affinities for Glu, and different sites of localization in regions of the brain or the synaptic region of neurons [9]. The overall influence that the small changes in expression of *Gria*'s, *Grm*'s and *Grik2 *may have on synaptic activity and neuronal integrity would be difficult to predict.

Other important molecular determinants of axon, dendrite and synapse growth included genes that were up-regulated in *Glud1 *Tg mice and are listed under the category of *Nervous system development *in Table 1. Among them were the ephrin type A receptor genes *Epha3, Epha4, Epha6*, and *Epha7*. Ephrin and the ephrin type A receptors are known to be particularly important in the development of the small protrusions that project from the shafts of the dendritic processes of neurons, the spines. Dendritic spines represent the post-synaptic elements of neuronal synapses and are extremely important in the establishment and function of excitatory synapses. Spine-associated synapses are involved in the physiological processes related to learning and memory [41]. Spine growth and synapse formation is also regulated by neurotrophic factors, such as Brain-Derived Neurotrophic Factor (BDNF), and the respective receptors for these neurotrophic factors, such as NTRK2 and NTRK3 [42,43].

Cytoskeleton organization and formation of focal adhesions also play important roles in the elongation of neurites (axons or dendrites) and synapse formation. Gene transcriptional activity for these cellular structures was activated in the hippocampus of Tg mice as revealed by the up-regulation of genes in the GO category of *Cytoskeleton and focal adhesion *and in the bio-function *Cellular assembly and organization *(Tables 1 and 2). Included among these up-regulated genes were *Anp32A *(Acidic Leucine-Rich Nuclear Phosphoprotein 32A), *Egr2 *(Early Growth Response 2) [44], *Ptk2b*, *Pitpnm2 *(coding for Phosphatidylinositol Transfer Protein Neuronal Membrane 2, a PTK2B-associated protein), and *Enc1 *(Ectodermal-Neural Cortex 1) [45]. *Anp32A *binds to a microtubule-associated protein during neurite formation or elongation [46], *Egr2 *regulates myelin sheath formation during axon development [44], *Ptk2b *and *Pitpnm2 *are involved in neurite elongation and synapse formation [47], and *Enc1 *is involved in neuronal differentiation [45]. Many of the same genes also appeared under the GO category and IPA bio-function of *Nervous system development *(Table 1), as would be expected for gene products that regulate the differentiation and growth of neurons during early development.

The GO category of *mRNA Processing *and the IPA bio-function of *RNA Post-transcriptional modification *contained many up-regulated genes in the Tg mouse hippocampus (Tables 1 and 2), including those for RNA editing, RNA binding and transport, and pre-mRNA splicing. Of particular interest among these genes was the gene *Syncrip*, the protein product of which has been identified to be associated with dendrites of neurons where it assists localized protein synthesis near synapses, possibly in response to incoming stimuli in neurons [48,49]. The up-regulation of *Syncrip *and other mRNA processing genes was indicative of increased trafficking and translation of mRNA's in the dendritic processes of *Glud1 *hippocampal neurons. This up-regulation of gene expression could be viewed as part of the pattern of neurite growth and differentiation described above.

The transcriptomic changes on synapse formation and neurite growth described in the present study of *Glud1 *Tg mice likely represent compensatory neuronal responses to chronic mild Glu hyperactivity. It is, of course, quite possible that the changes observed in 9-mo-old mouse hippocampi were the result of abnormal neural circuit development in Tg mice during embryonic or fetal development. Although such a scenario may be difficult to disprove, it should be pointed out that similar transcriptomic analyses at a very early age (10 days post-birth), in the same brain region as that analyzed in the present study, showed that the hippocampus of Tg mice at this young age is very similar to that of wt mice in terms of transcriptomic pattern (Wang, X., Hui, D., Michaelis, E.K., unpublished observations). These findings would indicate that the moderate Glu hyperactivity in the *Glud1 *Tg mice did not affect early brain development. Therefore, while exposure to moderate Glu hyperactivity during embryonic and fetal brain development might lead to small alterations in neural circuitry, these do not translate to the significant transcriptomic changes reported here.

### Gene network for neurite outgrowth and elongation

**Figure 2:**
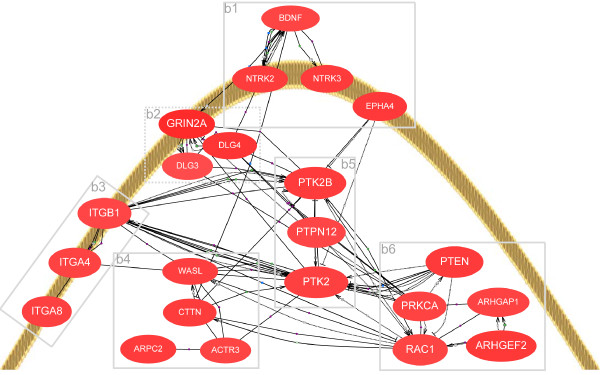
**Network of genes for neuritogenesis and neurite elongation**. A neurite growth cone and genes related to neuritogenesis and neurite elongation are shown. These genes are grouped on the basis of their functions and these gene groups are marked by dotted-line boxes. The function groups in these boxes are: b1 - growth factor and ephrin receptors; b2 - Glu receptor and scaffolding genes; b3 - extracellular matrix interacting genes; b4 - actin-organizing genes; b5 - focal adhesion complex genes; and b6 - signal transduction (kinase, phosphatase, small GTPase-binding and activating) genes. The lines between genes indicate their interactions based on previously published experimental data (multiple interaction types, such as activation, inhibition, binding, etc., exist in the network). All genes in this network were up-regulated in the *Glud1 *neurons, indicative of neuronal efforts to re-grow neurites in response to neurite loss observed in Tg mouse neurons. PTK2B, a major focal adhesion kinase, is a hub node in this network, indicating that it is one of the key gene players in neuritogenesis and neurite elongation.

In order to elucidate how genes involved in neurite outgrowth and elongation interact to promote neuritogenesis in the *Glud1 *mouse brain, a gene network was constructed for these genes and their partners (Figure 2). Key players in this network include proteins involved in focal adhesion of cell membranes to the extracellular matrix, the focal adhesion kinases [47], activators of the organization of cytoskeletal elements, the Rho GTPases, and cytoskeletal element organizing-proteins, actin and actin-associated proteins [43]. The information on gene-gene interactions displayed in this network was based on experimental evidence collected from previously published literature reports.

During neurite outgrowth, extracellular growth and guidance signals bind to their receptors on the distal end of the neurites (i.e., the growth cone) and such binding triggers downstream intracellular signal cascades that induce neurite elongation. BDNF is one of such upstream signals for neurite outgrowth and its transcription was modestly up-regulated in the Tg animals. The genes for two cell surface receptors of BDNF, *Ntrk2 *and *Ntrk3*, were significantly up-regulated. Other cell membrane receptors in this network that interact with the extracellular matrix during neurite elongation, include members of the integrin family, and several of those genes were also up-regulated. Integrins contain one α-subunit and one β-subunit; genes coding for both subunits were up-regulated, including *Itga4*, *Itga8*, and *Itgb1*.

Neuritogenesis requires efficient organization and regulation of the cytoskeleton in the growth cone region. Actin filament re-organization can be brought about by the actin-related protein complex 2/3 (*Arpc2*), which was up-regulated in *Glud1 *mice. The expression of several other genes whose products interact with actin filaments or microtubules in the growth cone was also up-regulated in *Glud1 *mouse hippocampus, including *Actr3 *(Actin-Related Protein 3), *Cttn *(Cortactin), and *Wasl *(Wiskott-Aldrich Syndrome-Like). PTK2B is also involved in actin reorganization within the growing ends of neurites [50].

The up-regulation of genes in this network, collectively, pointed in the direction of enhanced neurite outgrowth and elongation in neurons of *Glud1 *brain. From the constructed network, it was also clear that PTK2B is one of the most connected nodes and a central hub in the network. In the dendrites of neurons, PTK2B protein is associated with NMDA receptors and is involved in long-term changes of synaptic activity [51-54]. The role of NMDA receptors in synapse growth was described above. As captured in the network, genes for partners of PTK2B were also up-regulated in *Glud1 *hippocampal neurons, including the protein kinase *Prkca *and the small GTPase *Rac1 *(RAS-Related C3 Botulinum Substrate 1).

### Immunohistochemical characterization of PTK2B and phospho-PTK2B levels in hippocampal neurons

To initiate neurite outgrowth and elongation, PTK2B needs to be translocated from cytosol to the plasma membrane. This translocation depends on its biochemical activation via auto-phosphorylation on Tyr ^402 ^(PTK2BpY402), which is further dependent on the state of Ca^2+ ^signaling in neurons [55]. Excess [Ca^2+^]_i _in neurons subjected to increased Glu activity in *Glud1 *mice, may cause increases in PTK2BpY402 levels and lead to subsequent PTK2B translocation to the plasma membrane of neurons. To determine whether the events associated with PTK2B-mediated neurite growth are indeed more pronounced under the conditions of chronically increased Glu activity in brain, we probed for evidence of changes in the phosphorylation status of PTK2B, as well as for changes in total protein levels of PTK2B in *Glud1 *mice.

**Figure 3:**
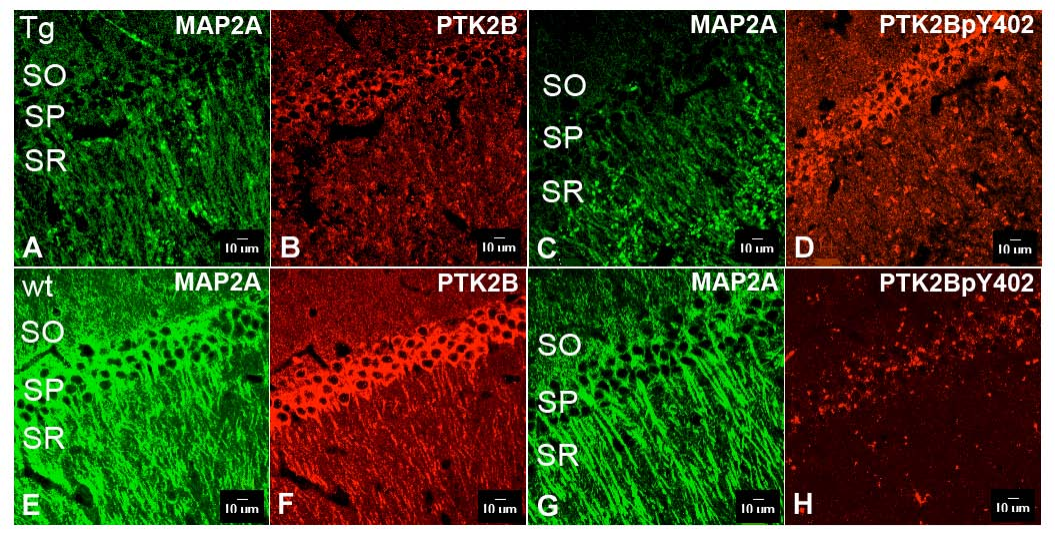
**Up-regulation of PTK2B detected by immunolabelling in the hippocampal CA1 region of *Glud1 *Tg mouse brain**. Antibodies against PTK2B, phosphorylated PTK2B (PTK2BpY402), and MAP2A were used to label reactive neurons in the hippocampal CA1 region of 11 mo *Glud1 *(A-D) and wt (E-H) mice. Immunolabelled sections were examined under confocal microscopy as described under Methods. Compared with hippocampus neurons in wt mouse, those in the *Glud1 *Tg mouse exhibited a substantial decrease in dendritic labelling by MAP2A (see also text). On the other hand, labelling by PTK2BpY402 in Tg mouse hippocampus appeared to be higher than that observed in wt mice. After normalization of PTK2BpY402 immunoreactivity by expressing it as a ratio of PTK2BpY402 to MAP2A labelling, the levels of PTK2BpY402 were significantly higher in the *Glud1 *Tg neurons (see also text), a possible sign of increase in neurite growth in neurons of Tg mice. SO, *stratum oriens*; SP, *stratum pyramidale*; SR, *stratum radiatum*. Scale bars: 10 μm.

Immunohistochemical labelling with anti-PTK2B pan-antibodies and selective antibodies against the phosphorylated form of PTK2B was performed in 10-12 mo-old mice, an age very close to the 9 mo-old mice used for the microarray analyses. Labelling with the pan-antibodies to PTK2B was strong in cell bodies and dendrites of hippocampal CA1 pyramidal neurons of wt mice, and relatively strong in *Glud1 *mice (e.g., Figure 3B, F). The cell bodies and dendrites of neurons in the same sections were also labelled by antibodies to microtubule-associated protein 2A (MAP2A), a protein that is known to be expressed in neuronal cell bodies and dendrites. As we reported previously [14], we observed lower levels of MAP2A labelling in dendrites of the CA1 region of the hippocampus of *Glud1 *mice as compared with the same region in wt mice (e.g., Figure 3A, E). Densitometric analyses of three fields of equal areas (3128 *μ*m^2^/field) in the CA1 *stratum radiatum *dendrite region from three hippocampus sections from three *Glud1 *and three wt mice, revealed significant decreases in MAP2A labelling in the Tg mice (MAP2A = 26.51 ± 2.56, mean ± SEM, for *Glud1*; 37.83 ± 1.31, for wt mice; *P *= 0.029, One-way ANOVA, pair-wise comparisons Student-Newman-Keuls). Yet, despite the loss of labelling by MAP2A, PTK2B labelling appeared to be well preserved in the dendrites of neurons from the *Glud1 *mice as revealed by the lack of significant differences in the same fields of the *stratum radiatum *of the CA1 region from the same sections of *Glud1 *and wt mouse hippocampus as those analyzed for MAP2A labelling (PTK2B = 43.72 ± 4.21, for *Glud1*; 41.32 ± .03, for wt; n = 3 Tg and 3 wt mice; *P *= 0.69, ANOVA).

In addition to the PTK2B labelling in dendrites, the levels of the phosphorylated form of PTK2B, PTK2BpY402, were also well preserved in the CA1 region of the hippocampus from *Glud1 *mice (Figure 3D, H). Once again, the labelling by MAP2A antibodies (e.g., Figure 3C, G) in these same sections from *Glud1 *mice was significantly lower than that in wt mice (MAP2A = 22.29 ± 2.66 for *Glud1 *mice; 32.88 ± 3.01, for wt mice; n = 3 Tg and 3 wt mice; *P *= 0.039, ANOVA). In order to standardize the labelling by PTK2BpY402 to the loss of MAP2A labelling in dendrites of 10-12 mo-old Tg mice, we examined the ratio of labelling (pixel density) by anti-PTK2BpY402 to that of anti-MAP2A. This ratio was significantly higher in the *Glud1 *mice (*Glud1 *= 1.22 ± 0.38; wt = 0.37 ± 0.02; n = 3 Tg and 3 wt mice; *P *= 0.046, ANOVA).

To determine whether these observations were consistent across different ages of Tg and wt mice, we also examined the same brain regions in three pairs of younger (5 mo-old) and in three pairs of older (19-20 mo-old) *Glud1 *and wt mice, using identical methodologies as described above. Two-way ANOVA of the ratios of PTK2BpY402 to MAP2A labelling (pixel density) in the *stratum radiatum *of the CA1 region of 5, 10-12, and 19-20 mo-old mice indicated a significant effect of the transgene *Glud1 *when all ratios across all ages in *Glud1 *mice were compared with those of the wt (*P *= 0.002). Pair-wise comparisons (Student-Newman-Keuls method) revealed significant differences in the ratios between Tg and wt mice of 10-12 mo of age (*P *= 0.013) and of 19-20 mo of age (*P *= 0.028). Although there was a trend of increasing PTK2BpY402 to MAP2A ratios with advancing age in both wt and Tg mice, there was no significant age effect detected (*P *= 0.236). Overall, the immunohistochemical characterization of PTK2B and phospho-PTK2B levels in hippocampal neurons indicated that not only protein levels but also biochemical activation (i.e., phosphorylation) of PTK2B were significantly increased in the Tg mouse hippocampus.

**Figure 4:**
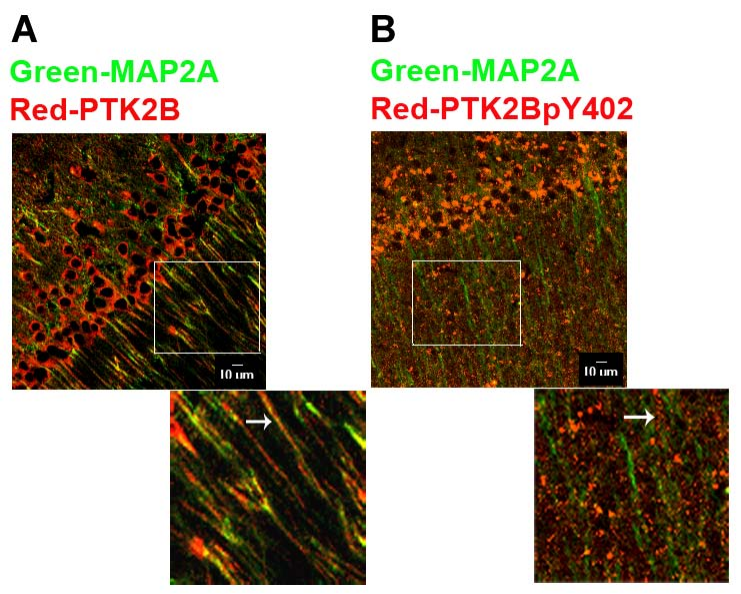
**Localization of PTK2B and PTK2BpY402 in dendrites of CA1 hippocampus neurons in Tg mice**. Labelling with antibodies to PTK2B, PTK2BpY402, and MAP2A were performed exactly as in Figure 3. The labelled sections were imaged using confocal microscopy and are shown as super-imposed images of PTK2B and MAP2A (A) or PTK2BpY402 and MAP2A (B). Sections were obtained from 20 mo-old mice. Enlarged images of dendrite regions from the *stratum radiatum *enclosed by white squares in A and B are presented below the respective images. Note the relatively uniform distribution of PTK2B in dendrites of the CA1 region as compared to the large puncta of immunolabelling by anti-PTK2BpY402. Arrows point to the two types of labelling by anti-PTK2B and anti-PTK2BpY402 antibodies along the path of a dendrite in CA1 *stratum radiatum*. Bar: 10 μm.

The *in situ *labelling of PTK2B and PTK2BpY402 revealed the differential pattern of distribution of the activated and non-activated forms of this key kinase. Double immune labelling with MAP2A and PTK2B showed a relatively smooth, continuous pattern of distribution and co-localization of both proteins in dendrites, although the PTK2B labelling appeared to be more superficial than that of MAP2A in the dendrite shafts (Figure 4A). On the other hand, PTK2BpY402 was accumulated in small or large puncta, some of them projecting off the surface of CA1 neuronal dendrites (Figure 4B). These phospho-PTK2B accretions might represent areas of growth of new spines, new dendrite branches, or new synapses.

### Increased Ca^2+ ^signalling, Ca^2+ ^extrusion from cells, and Glu transport into glial cells

As mentioned above, Glu activation of its receptors leads to increased Ca^2+ ^influx into neurons and may initiate a series of injurious events for neurons. In addition, the processes of spine and synapse formation in brain neurons are regulated by intracellular Ca^2+ ^levels [56]. A rise in intracellular Ca^2+ ^concentration to a low level, leads to elongation or growth of spines, whereas at higher levels causes spine retraction. Since increased release of synaptic Glu would likely lead to hyper-activation of its receptors and increased accumulation of intracellular Ca^2+^, it might be expected that neurons of the *Glud1 *mice would adapt to over-stimulation of Glu receptors by increasing systems that either pump Ca^2+ ^out of cells or sequester Ca^2+ ^into organelles. It is not surprising, therefore, that the GO category of *Ion transport and homeostasis *and the IPA canonical pathway of *Ca*^*2+ *^*Signalling *included the two key genes involved in Ca^2+ ^transport out of cells, *Atp2b2 *(ATPase, Ca^2+ ^Transporting, Plasma Membrane 2) and *Slc8a2 *(Solute Carrier Family 8 [Sodium/Calcium Exchanger], Member 2) (Tables 1 and 2). In addition to the genes whose function is the regulation of intracellular Ca^2+ ^levels, there were several genes whose expression was up-regulated and whose function is the modulation of intracellular signalling mediated by Ca^2+^, such as *Camk2a*, *Camk2b*, and *Ppp3ca *(Protein Phosphatase 3, Catalytic Subunit, Alpha Isoform). The proteins coded by these three genes have important functions in synaptic transmission and long-term changes in synaptic activity.

Cells in the hippocampus of *Glud1 *mice made, apparently, compensatory changes not only in the handling of Ca^2+ ^but also in the elimination of excess Glu released by neurons in the brain of Tg mice. The major Glu transporters that remove Glu from the brain synaptic cleft are SLC1A2 (Solute Carrier Family 1 [Glial High Affinity Glutamate Transporter], Member 2) and SLC1A3, another glial high affinity Glu transporter. SLC1A2 is the predominant transporter expressed in the plasma membrane of hippocampus glial cells [57]. The transcription of *Slc1a2 *was highly (2.2-fold) up-regulated in *Glud1 *mice (Additional file 1). In addition, the transcription of the gene *Slc1a1 *for the neuronal Glu transporter was moderately (1.28-fold) increased in neurons of *Glud1 *mice. In separate studies, we found that the levels of the SLC1A1 protein were increased in nerve ending particle (synaptosome) prepared from the brains of 12-mo-old Tg mice (1.39-fold) over those of wt mouse brain. Although the differences between *Glud1 *and wt mice in gene and protein levels were not large, transport of radioactive Glu ([^3^H]Glu) into nerve ending particles by, primarily, the neuronal Glu transporters was significantly increased: V_max _for [^3^H]Glu transport in *Glud1 *mouse particles was 5.7 ± 0.57 pmol•mg protein^-1^•min^-1 ^and for the wt was 3.4 ± 0.46 pmol•mg protein^-1^•min^-1 ^(*P *= 0.013; n = 3). The increased gene expression of Glu transporters together with the increases in protein and transport activity were congruent with the idea of a compensatory response in Tg mice that would clear synapses of excess extracellular Glu in the brain.

### Correlation of GeneChip data with quantitative PCR and immunoblot assays

**Figure 5:**
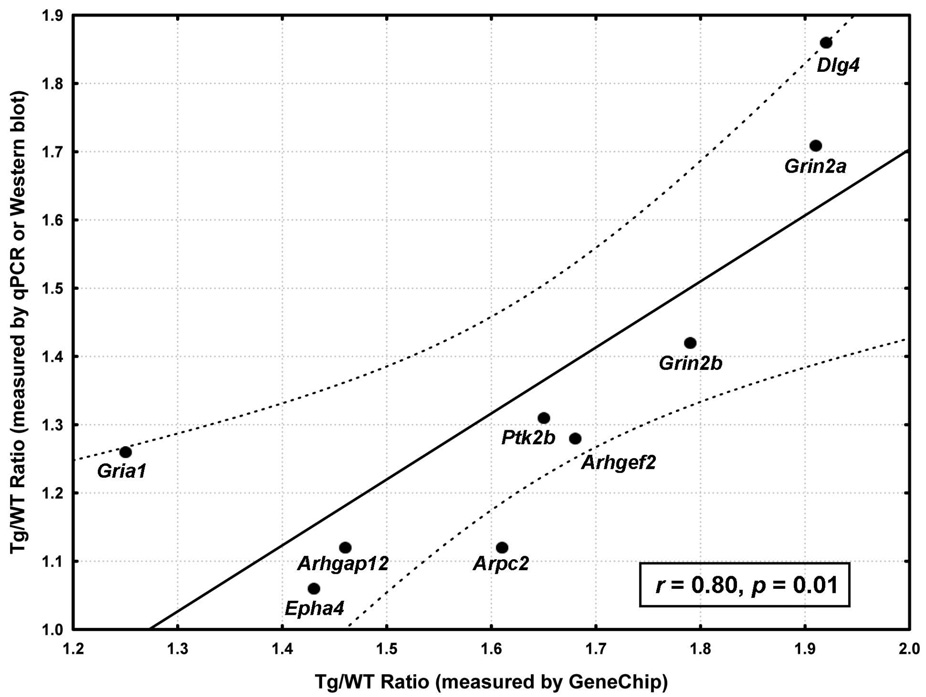
**Correlation of GeneChip data with real-time quantitative PCR or immunoreactivity data of nine important genes with significantly altered expression in the *Glud1 *mice**. To validate the GeneChip data, confirmatory data were collected by both real-time quantitative PCR and Western blot analyses. Three replicate measurements were performed on each gene (or protein) and the mean value is shown here. Real-time quantitative PCR was used for the measurements of *Arhgap12*, *Arhgef2*, *Arpc2 *and *Epha4*, while Western blots were used for the products of *Dlg4*, *Gria1*, *Grin2a*, *Grin2b *and *Ptk2b*. The Pearson correlation coefficient (*r*) between the GeneChip and confirmatory data, and the estimated *p *value, are shown at the bottom. The best-fit linear regression line (solid line) and the estimated 95% confidence limits (dashed lines) are also shown.

To validate the GeneChip-derived gene expression data, we conducted real-time quantitative PCR and immunoblot analyses of several key genes or their protein products. The obtained results indicated a strong correlation of these data with the data from microarray analyses (Figure 5). This correlation was significant, with the Pearson correlation coefficient (*r*) being equal to 0.80 and the estimated *P *value equal to 0.01.

## Conclusions

In our previous studies, we have shown that over-expression of *Glud1 *and the resultant moderate excess Glu formation and synaptic release, were correlated with neuronal, dendritic spine, and nerve ending losses in select regions of the brains of Tg mice, such as the CA1 region of the hippocampus. The selective nature of neuronal damage in *Glud1 *mice probably contributes to their survival into early aging, thus affording the opportunity to examine the effects of *Glud1 *over-expression and excess Glu formation and release on molecular and cellular pathways in brain across the lifespan.

Aging in human brain is associated with the selective loss of synapses and neurons that are glutamatergic and occurs in the absence of widespread loss of neurons or synapses throughout the brain [19,21,58]. The neuronal populations in human brain that are susceptible to injury are some of the same populations of neurons that die in increasing numbers with advancing age in *Glud1 *Tg mice [14]. In this respect, the *Glud1 *Tg mice are, potentially, a good model of the selective synapse and nerve cell losses that are associated with advancing age in humans. Several studies have shown that baseline extracellular levels of Glu in brain, as well as the increases in Glu release following depolarization of neurons, are 75 to 100% greater in the brains of aged rodents as compared with those of young organisms [59,60]. The age-associated increases in extracellular Glu are thought to occur because of some loss of function of Glu transporters during aging [61-64].

The *in vivo *effects of life-long increases in Glu formation and depolarization-induced release of Glu on neuronal structure, function and adaptive molecular responses of neurons and glia have not been characterized previously. Most studies have examined the cellular and molecular responses of neurons to the toxicity produced following acute exposure to excess Glu or NMDA [65,66]. As mentioned in a previous publication [14], the neuronal damage caused by chronic Glu hyperactivity under neuropathological conditions cannot be easily reproduced by *in vitro *acute Glu treatments of neurons. Acute exposure of neurons to Glu does not allow for the determination of long-term adaptive responses and repair efforts in affected neurons. In addition, acute exposure to high concentrations of Glu presents difficulties in dissociating molecular responses representing recovery from neuronal injury from those related to cell death. Mechanistically, acute Glu treatments lead to increased transport of ions (Ca^2+ ^, Na^+^, K^+ ^and Cl^-^) [13,67,68], dysfunction in calcium-mediated signal transduction [69-73], oxidative stress [74-77], cytoskeletal alterations [78-80], changes in gene transcription through altered activities of the transcription factors NF-κB, AP-1 or CREB [81-86], and cell death (including apoptosis, necrosis and autophagy) [12,87-89]. Acute Glu treatments also bring about significant changes in mitochondrial function, including decreased ATP synthesis, depolarization, and structural collapse [90,91]. The moderate, chronic increases in Glu levels and Glu release in the *Glud1 *Tg mice appear to produce molecular responses that may counteract in neurons the adverse effects of Glu hyperactivity, such as excess Ca^2+ ^accumulation, altered mitochondrial metabolism, energy depletion, and neuronal death. Although we cannot totally exclude the possibility of minor mitochondrial changes in *Glud1 *Tg neurons, our transcriptomic analysis did not show observable signs of significant mitochondrial dysfunction at the age probed (9 month).

Compared with acute Glu treatments, a distinctive characteristic of chronic moderate Glu hyper-activity in neurons of *Glud1 *Tg mice is the apparently compensatory response of neurite regrowth and synapse formation. Other responses, such as chronic inflammatory response, are also unique to the *Glud1 *Tg mouse model. Because chronic inflammation is an important aspect of brain aging and many neurodegenerative conditions [92-94], these responses in *Glud1 *Tg mice make this mouse a more useful model of chronic brain neurodegenerative conditions than the acute Glu treatment models of neurotoxicity.

Selective neuronal damage in the brain of *Glud1 *mice and the survival of these mice to an age greater than 21 months, distinguishes them from null mutant mice for the Glu transporters or for the *Tsc-1 *gene. These other animal models of high Glu concentrations in the extracellular space are characterized by disruption of normal brain development, lethal spontaneous seizures, neuronal death in many regions of the brain, and dramatically shortened lifespan [15-18]. Therefore, they are not suitable for probing the effects of chronic, moderate increases in Glu synaptic release on neurons. To our knowledge, there are no functional genomics studies that have been performed on any of these animal models. While it is possible that the toxicity to neurons caused by tonically high levels of extracellular Glu in these animals may share some commonalities with those reported here for the *Glud1 *Tg mice, it is nevertheless evident that the *Glud1 *Tg model is unique in capturing the effects of moderate increases in Glu activity during brain aging without the extensive and early developmental neuropathology observed in other animal models.

In summary, chronic exposure of brain neurons and glial cells to increased extracellular release of the neurotransmitter Glu led to up-regulation of a constellation of genes in hippocampal cells. The main functional categories and molecular pathways of the genes that were over-expressed in the *Glud1 *mice were those of cell responses to oxidative stress, cell injury, and inflammation, as well as those of nervous system development, neurite growth, and synaptic transmission. The overall transcriptomic profile of *Glud1 *mice was indicative of apparent compensatory responses that were either protective against stress or promoting neuronal recovery. A key gene in the pathway of neurite growth and synapse formation was identified in this study as being that of the kinase *Ptk2b*. We determined that the gene and the activated form of this protein, the phosphorylated PTK2B, were up-regulated in *Glud1 *Tg hippocampal neurons that are normally susceptible to the injury that results from hyper-glutamatergic activity. The *Glud1 *over-expressing mouse does show an acceleration in the rate of aging-associated changes observed in normal brain [14], yet some of the neurobiological functions and molecular pathways altered in the transgenic mouse brain differ significantly from those altered during aging. Whereas *Stress response, DNA repair*, *Inflammation*, and *Transcription *categories of genes are up-regulated in *Glud1 *mice and the aged brain, the categories of *Synaptic transmission, Ca*^*2+ *^*homeostasis & signalling*, *Vesicular transport*, and *Cell signalling *are up-regulated in *Glud1 *mice but down-regulated in the aging human brain [95]. We conclude, therefore, that gene expression in 9 mo-old *Glud1 *mice may reflect two molecular and cellular responses to Glu hyper-activity, an accelerated form of aging at the cellular and transcriptomic level, and compensatory responses to Glu hyper-activity that lead to neuronal recovery of growth and function. The present study has focused on the molecular pathways that may be affected by the over-expression of *Glud1 *in neurons in young adult mice. Future studies will explore changes in these molecular patterns with advancing age.

## Methods

### Animals used for transcriptomic analyses

Nine month-old male *Glud1 *Tg mice were used in the transcriptomic study. The procedure for generating the Tg mice was described in detail previously [14]. Age-matched, wild-type, male mice of the same genetic background (C57BL/6) were used as the controls. A total of three *Glud1 *Tg and three control mice were used. All animals were housed in a 12 hour light/dark cycle with food and water *ad libitum*. All animal procedures were performed in accordance with guidelines established by the University of Kansas IACUC. Briefly, the animals were anesthetized, hippocampi dissected out from the brains, flash frozen in liquid nitrogen, and stored at -80°C prior to total RNA extraction.

### RNA extraction and microarray data generation

Total RNA samples were extracted from the dissected hippocampi using the Qiagen RNeasy^® ^Mini Kit (Qiagen, Valencia, CA, USA). To prepare targets for subsequent GeneChip hybridization, One-Cycle cDNA Synthesis Kit from Affymetrix (Santa Clara, CA, USA) was used and the manufacturer's instructions were followed. The Affymetrix GeneChip Mouse Genome 430 2.0 arrays, which are designed to interrogate expression of over 39,000 mouse gene transcripts, were used for the hybridization. Subsequent washing and staining steps were performed on a GeneChip Fluidics Station 450 and the chips were scanned on a GeneChip Scanner 3000 (Affymetrix). Instrument control and data collection were carried out using GeneChip Operating Software (GCOS, ver 1.1.1). In order to minimize experimental variability, all steps in tissue dissection, RNA isolation and microarray operation were performed by a single investigator. The quality and quantity of the original RNA samples and of the cRNA probes generated for array hybridization were determined with an Agilent 2100 Bioanalyzer (Agilent Technologies, Palo Alto, CA, USA), and a NanoDrop ND-1000 Spectrophotometer (Thermo Scientific, Wilmington, DE, USA). The microarray data generated from all chips met the quality control criteria set by Affymetrix, including low background and noise, positive detection of QC probesets such as bioB, percentage of genes called present in normal range (generally between 40-60%), similar scaling factors across all chips, and acceptable 3'/5' ratios. All microarray data were deposited in NCBI's Gene Expression Omnibus (GEO), with series accession number GSE11419.

### Transcriptomic data analysis

The generated GeneChip CEL data were normalized by the Robust Multiarray Average (RMA) algorithm [96]. Prior to the identification of genes showing significant differential expression patterns, probesets that were detected to be non-expressed, i.e., those with absence calls, were filtered out and excluded from further analysis. In addition, probesets that are designed solely for the purpose of chip quality control (monitoring hybridization target preparation and array hybridization), i.e., those with prefix AFFX, were also filtered out. These filtering steps were employed in order to reduce the number of comparisons in subsequent analyses and thus reduce the FDR. SAM (Significance Analysis of Microarrays), a supervised learning software for genomic expression data mining [97], was used to identify genes showing significant differential expression patterns. Cyber-T analysis [98] was used to confirm expression patterns of differential genes identified by SAM. Three criteria were used to determine statistically significant differential expression of genes between Tg and wt mice: Bayesian *P *value ≤ 0.05, fold change ≥ 1.3, and FDR ≤ 1%. Hierarchical clustering of the samples (condition tree) was performed using the software GeneSpring GX ver7.3 (Agilent Technologies). Gene Ontology (GO) analysis was conducted with GO-elite, a MAPPFinder analysis tool [99]. The gene annotation and GO-elite databases used for the analyses were those of March 2008. Hypergeometric distribution was used and a Z score > 1.96 or < -1.96 and a permute *P *value < 0.05, were considered to be statistically significant. Comparative analyses on bio-function and canonical pathways of the identified differentially expressed genes were conducted using IPA (Ingenuity Systems, Redwood City, CA, USA). For these analyses, Fischer's exact test was used to calculate the *P *values. The reconstruction of the neuritogenesis network was performed using Pathway Studio (Ariadne Genomics, Rockville, MD, USA). The ResNet database used for the reconstruction was of January 2008.

### Real-time quantitative PCR

The remainder of the hippocampal total RNA extracts not used in the microarray studies was used for real-time PCR. The RNA was reverse-transcribed to cDNA using oligo(dT) primers. Taqman Gene Expression Assay reagents (Applied Biosystems, Foster City, CA, USA) were employed in the real-time PCR using an Applied Biosystems 7500 Fast Real-Time PCR System in fast mode. The PCR thermal cycling conditions were: an initial step of 20 sec at 95°C, followed by 40 cycles of 95°C for 3 sec, and 60°C for 30 sec. Quantification of the target genes was based on the relative standard curve method [100]. The measurements were standardized against the gene for neuron-specific enolase (*Nse*, or *Eno2*) as its ratio between the *Glud1 *and wild-type mice was the one closest to unity (0.98).

### Immunoblot assays

For immunoblot studies, proteins were first extracted from hippocampi dissected from the same animals as those used for the microarray study, i.e., three 9-mo-old *Glud1 *Tg and three age-matched wt male mice. The protein extracts were subjected to sodium dodecyl sulfate polyacrylamide gel electrophoresis (SDS-PAGE) and immunoblotting as described previously [101]. Anti-DLG4, -GRIA1, -GRIN2A, -GRIN2B, and -PTK2B antibodies were used at 1:1000 to 1:2000 dilutions. Relative levels of expression were estimated by densitometry.

### Immunohistochemical analysis of PTK2B and PTK2BpY402 levels

Three pairs of wt and Tg male mice from three age groups were used for the immunohistochemical studies (total 9 wt and 9 Tg mice). The age groups were: 5 mo-old, 10-12 mo-old, and 19-20 mo-old wt and Tg mice. Brains from these animals were dissected out and immersion-fixed in 4% paraformaldehyde-phosphate buffered saline (PBS) for 72 hr at 4°C. Following fixation, the brains were cryoprotected (incubation in 30% sucrose/PBS), frozen by exposure for 30 sec to 2-methyl-butane at liquid N_2 _temperatures, and coronal sections (24 μm thickness) cut in a cryotome. The sections were then transferred onto gelatin-coated glass slides. The sections were blocked with 3% (w/v) gelatin in PBS (1 hr at 37°C), treated with 0.1% Triton X-100 in PBS (15 min at 23°C), and reacted (overnight at 4°C, then 1 hr at 23°C) with the respective primary antibodies. Polyclonal PTK2B antibody was used at a dilution of 1:300 and polyclonal anti-PTK2BpY402 at 1:250. Each section was also exposed to monoclonal anti-MAP2A at 1:1,000 dilution. Following rinsing of the sections in PBS, they were incubated (2 hr at 37°C) with fluorescent dye-labelled secondary antibodies and processed as described [101,102]. The sections were rinsed in PBS (5 min at 23°C), mounted in 70% glycerol, and viewed using a Zeiss LSM 510 confocal microscope [101]. For densitometric measurements and quantification of pixel densities of anti-PTK2B, anti-PTK2BpY402, and anti-MAP2A immunolabelled sites in neuronal dendrites in the *stratum radiatum *of the hippocampus CA1 region, measurements of pixel densities were obtained from within three equal-sized areas (3128 *μ*m^2^) from three sections from each mouse hippocampus using the Photoshop program. All data were analyzed using either One-Way or Two-Way ANOVA with *post hoc *multiple pairwise comparisons (Student-Newman-Keuls method). Significance levels were *P *= 0.05.

## Abbreviations

CNS: central nervous system; DEG: differentially expressed genes; FDR: false discovery rate; Glu: glutamate; *Glud1*: glutamate dehydrogenase 1 gene; GO: Gene Ontology; IPA: Ingenuity Pathway Analysis; PTK2B: protein tyrosine kinase 2 beta; SAM: Significance Analysis of Microarrays; Tg: transgenic; wt: wild type.

## Authors' contributions

XW and EKM conceived of the study and drafted the manuscript. XW also carried out the RNA extraction, GeneChip data generation, bioinformatic data analyses, and real-time quantitative PCR confirmation of the GeneChip data. EKM was also involved in the interpretation of the transcriptomic data and coordinated the study. XB developed the *Glud1 *transgenic mice, collected the brain samples for the transcriptomic analysis, and participated in the real-time quantitative PCR analysis. RP conducted the immunohistochemical analysis. AA carried out the immunoblot assays. All authors read and approved the manuscript.

## Supplementary Material

Additional file 1 **Prominent up- and down-regulated genes in Glud1**. A list of prominently up- and down-regulated genes associated with chronic glutamate hyperactivity in the *Glud1 *mice. Click here for file

Additional file 2 **All differential probesets in Glud1**. Complete list of significantly up- and down-regulated probesets identified in this study. The 707 up- and 311 down-regulated probesets are listed in two separate data sheets of this Excel file. The probeset IDs are listed along with corresponding gene symbols, gene titles, fold changes and significance *P *values (calculated from Cyber-T test). Click here for file

Additional file 3 **IPA bio-functions and genes in Glud1 Tg**. Complete list of statistically significant bio-functions and associated genes from the IPA analysis. The bio-functions are reported in Figure 1D. Click here for file

Additional file 4 **IPA pathways and genes in Glud1 Tg**. Complete list of statistically significant canonical pathways and associated genes from the IPA analysis. The canonical pathways are reported in Figure 1E. Click here for file
