# Supplementary material for: Transcriptomic responses in mouse brain exposed to chronic excess of the neurotransmitter glutamate
Source: BMC Genomics. 2010 Jun 7;11:360. doi: 10.1186/1471-2164-11-360 (PMC2896956; doi:10.1186/1471-2164-11-360)
Supplement: Additional file 1 — Prominent up- and down-regulated genes in Glud1. A list of prominently up- and down-regulated genes associated with chronic glutamate hyperactivity in the Glud1 mice. [file 1471-2164-11-360-S1.DOC]

**Additional Table 1 – Prominent up- and down-regulated genes associated with chronic glutamate hyperactivity in the *Glud1* mice**

| **Affymetrix ID** | **Gene Symbol** | **Gene Title** | **Fold Change** | ***P**** |
| --- | --- | --- | --- | --- |
| ***Up-regulated*** | | | | |
| 1450407_a_at | *Anp32a* | acidic (leucine-rich) nuclear phosphoprotein 32 family, member A | 3.40 | 0.000119 |
| 1427683_at | *Egr2* | early growth response 2 | 2.73 | 1.22E-06 |
| 1427688_a_at | *Ptprs* | protein tyrosine phosphatase, receptor type, S | 2.28 | 7.84E-06 |
| 1459014_at | *Slc1a2* | solute carrier family 1 (glial high affinity glutamate transporter), member 2 | 2.20 | 0.000774 |
| 1443188_at | *Ube2w* | ubiquitin-conjugating enzyme E2W (putative) | 2.20 | 1.06E-06 |
| 1423100_at | *Fos* | FBJ osteosarcoma oncogene | 2.06 | 7.69E-05 |
| 1435493_at | *Dsp* | desmoplakin | 1.95 | 2.4E-05 |
| 1420964_at | *Enc1* | ectodermal-neural cortex 1 | 1.94 | 0.000309 |
| 1419581_at | *Dlg4* | discs, large homolog 4 (Drosophila) | 1.92 | 0.000507 |
| 1421616_at | *Grin2a* | glutamate receptor, ionotropic, NMDA2A (epsilon 1) | 1.91 | 0.001218 |
| 1444671_at | *Rasal2* | RAS protein activator like 2 | 1.89 | 0.000111 |
| 1420816_at | *Ywhag* | 3-monooxygenase/tryptophan 5-monooxygenase activation protein, gamma polypeptide | 1.87 | 4.02E-05 |
| 1430197_a_at | *Pitpnm2* | phosphatidylinositol transfer protein, membrane-associated 2 | 1.83 | 4.38E-05 |
| 1459826_at | *Kcnq2* | Potassium voltage-gated channel, subfamily Q, member 2 | 1.82 | 0.000251 |
| 1446712_at | *Ntrk2* | neurotrophic tyrosine kinase, receptor, type 2 | 1.81 | 1.5E-05 |
| 1421166_at | *Atrn* | attractin | 1.80 | 8.25E-05 |
| 1430980_a_at | *Eif4a1* | eukaryotic translation initiation factor 4A1 | 1.79 | 2.15E-06 |
| 1422223_at | *Grin2b* | glutamate receptor, ionotropic, NMDA2B (epsilon 2) | 1.79 | 0.000205 |
| 1437118_at | *Usp7* | ubiquitin specific peptidase 7 | 1.78 | 5.6E-05 |
| 1421789_s_at | *Arf3* | ADP-ribosylation factor 3 | 1.78 | 5.31E-05 |
| 1438069_a_at | *Rbm5* | RNA binding motif protein 5 | 1.78 | 0.00016 |
| 1418687_at | *Arc* | activity regulated cytoskeletal-associated protein | 1.75 | 1.25E-05 |
| 1449876_at | *Prkg1* | protein kinase, cGMP-dependent, type I | 1.75 | 0.000198 |
| 1446144_at | *Pex5l* | peroxisomal biogenesis factor 5-like | 1.75 | 7.2E-06 |
| 1431749_a_at | *Rasgrp1* | RAS guanyl releasing protein 1 | 1.73 | 2.19E-05 |
| 1438126_at | *Exoc5* | exocyst complex component 5 | 1.73 | 6.12E-05 |
| 1417752_at | *Coro1c* | coronin, actin binding protein 1C | 1.73 | 0.000196 |
| 1425885_a_at | *Kcnab2* | potassium voltage-gated channel, shaker-related subfamily, beta member 2 | 1.70 | 0.000175 |
| 1427646_a_at | *Arhgef2* | rho/rac guanine nucleotide exchange factor (GEF) 2 | 1.68 | 0.000548 |
| 1446224_at | *Hectd2* | HECT domain containing 2 | 1.66 | 0.000629 |
| 1425963_at | *Cabp7* | calcium binding protein 7 | 1.66 | 0.000114 |
| 1450442_at | *Add2* | adducin 2 (beta) | 1.66 | 0.000231 |
| 1442927_at | *Ptk2b* | PTK2 protein tyrosine kinase 2 beta | 1.65 | 0.000107 |
| 1422231_a_at | *Tnfrsf25* | tumor necrosis factor receptor superfamily, member 25 | 1.63 | 0.002307 |
| 1425061_at | *Wasf3* | WAS protein family, member 3 | 1.62 | 0.002678 |
| 1422044_at | *Ndst1* | N-deacetylase/N-sulfotransferase (heparan glucosaminyl) 1 | 1.61 | 0.004522 |
| 1442295_at | *Arpc2* | Actin related protein 2/3 complex, subunit 2 | 1.61 | 0.000378 |
| 1424893_at | *Ndel1* | nuclear distribution gene E-like homolog 1 (A. nidulans) | 1.58 | 0.000685 |
| 1447222_at | *Hspa12a* | heat shock protein 12A | 1.58 | 0.000805 |
| 1427306_at | *Ryr1* | ryanodine receptor 1, skeletal muscle | 1.57 | 0.000696 |
| 1425263_a_at | *Mbp* | myelin basic protein | 1.57 | 0.000153 |
| 1443125_at | *Trip12* | thyroid hormone receptor interactor 12 | 1.57 | 0.001926 |
| 1439479_at | *Lct* | lactase | 1.54 | 6.18E-05 |
| 1429607_at | *Trak2* | trafficking protein, kinesin binding 2 | 1.54 | 0.0019 |
| 1425805_a_at | *Usp12* | ubiquitin specific peptidase 12 | 1.54 | 0.004241 |
| 1432344_a_at | *Aplp2* | amyloid beta (A4) precursor-like protein 2 | 1.54 | 0.000705 |
| 1421199_at | *Dlg2* | discs, large homolog 2 (Drosophila) | 1.54 | 0.00965 |
| 1459894_at | *Iqgap2* | IQ motif containing GTPase activating protein 2 | 1.54 | 0.000528 |
| 1426043_a_at | *Capn3* | calpain 3 | 1.53 | 0.000472 |
| 1450319_at | *Gabrb2* | gamma-aminobutyric acid (GABA-A) receptor, subunit beta 2 | 1.52 | 0.002408 |
| 1431196_at | *Atp2c1* | ATPase, Ca++-sequestering | 1.52 | 0.000827 |
| 1450804_at | *Kif5c* | kinesin family member 5C | 1.51 | 0.001019 |
| 1450955_s_at | *Sort1* | sortilin 1 | 1.51 | 0.000207 |
| 1428054_at | *Slc8a2* | solute carrier family 8 (sodium/calcium exchanger), member 2 | 1.50 | 0.005133 |
| 1424332_at | *Rab40c* | Rab40c, member RAS oncogene family | 1.50 | 0.001746 |
| 1434763_at | *Orai2* | ORAI calcium release-activated calcium modulator 2 | 1.50 | 0.000151 |
| 1422072_a_at | *Gstm6* | glutathione S-transferase, mu 6 | 1.50 | 0.002571 |
| 1460307_at | *Akt3* | thymoma viral proto-oncogene 3 | 1.50 | 0.000655 |
| ***Down-regulated*** | | | | |
| 1416953_at | *Ctgf* | connective tissue growth factor | -3.07 | 1.33E-05 |
| 1423760_at | *Cd44* | CD44 antigen | -2.69 | 1.09E-07 |
| 1422860_at | *Nts* | neurotensin | -2.40 | 1.17E-06 |
| 1455304_at | *Unc13c* | unc-13 homolog C (C. elegans) | -2.27 | 2.47E-07 |
| 1451021_a_at | *Klf5* | Kruppel-like factor 5 | -2.03 | 2.49E-06 |
| 1424606_at | *Cplx3* | complexin 3 | -1.98 | 6.92E-06 |
| 1433607_at | *Cbln4* | cerebellin 4 precursor protein | -1.96 | 4.25E-06 |
| 1428379_at | *Slc17a6* | solute carrier family 17 (sodium-dependent inorganic phosphate cotransporter), member 6 | -1.94 | 3.72E-05 |
| 1449571_at | *Trhr* | thyrotropin releasing hormone receptor | -1.88 | 0.000341 |
| 1449368_at | *Dcn* | decorin | -1.83 | 5.56E-05 |
| 1435162_at | *Prkg2* | protein kinase, cGMP-dependent, type II | -1.77 | 8.29E-05 |
| 1422659_at | *Camk2d* | calcium/calmodulin-dependent protein kinase II, delta | -1.75 | 5.94E-05 |
| 1416158_at | *Nr2f2* | nuclear receptor subfamily 2, group F, member 2 | -1.73 | 0.00031 |
| 1441382_at | *Gpr101* | G protein-coupled receptor 101 | -1.72 | 0.000166 |
| 1435551_at | *Fhod3* | formin homology 2 domain containing 3 | -1.70 | 0.000284 |
| 1450700_at | *Cdc42ep3* | CDC42 effector protein (Rho GTPase binding) 3 | -1.70 | 0.000415 |
| 1460009_at | *Ier5* | immediate early response 5 | -1.70 | 0.001233 |
| 1455688_at | *Ddr2* | discoidin domain receptor family, member 2 | -1.68 | 0.000851 |
| 1437029_at | *Tacr3* | tachykinin receptor 3 | -1.67 | 7.92E-05 |
| 1417933_at | *Igfbp6* | insulin-like growth factor binding protein 6 | -1.66 | 0.000496 |
| 1422742_at | *Hivep1* | human immunodeficiency virus type I enhancer binding protein 1 | -1.65 | 8.44E-05 |
| 1434759_at | *Lrrtm3* | leucine rich repeat transmembrane neuronal 3 | -1.63 | 0.001694 |
| 1455324_at | *Plcxd2* | phosphatidylinositol-specific phospholipase C, X domain containing 2 | -1.62 | 3.76E-07 |
| 1435971_at | *Rims3* | regulating synaptic membrane exocytosis 3 | -1.62 | 7.98E-05 |
| 1434891_at | *Ptgfrn* | prostaglandin F2 receptor negative regulator | -1.62 | 0.000777 |
| 1448213_at | *Anxa1* | annexin A1 | -1.61 | 0.000868 |
| 1424902_at | *Plxdc1* | plexin domain containing 1 | -1.60 | 0.00062 |
| 1435790_at | *Olfm2* | olfactomedin 2 | -1.59 | 0.000103 |
| 1436661_at | *Dpp10* | dipeptidylpeptidase 10 | -1.58 | 0.000132 |
| 1437277_x_at | *Tgm2* | transglutaminase 2, C polypeptide | -1.58 | 0.001076 |
| 1451867_x_at | *Arhgap6* | Rho GTPase activating protein 6 | -1.58 | 0.001458 |
| 1438782_at | *Cntn4* | contactin 4 | -1.57 | 0.002807 |
| 1447669_s_at | *Gng4* | guanine nucleotide binding protein (G protein), gamma 4 subunit | -1.53 | 2.05E-05 |
| 1440484_at | *Unc5d* | unc-5 homolog D (C. elegans) | -1.53 | 0.001411 |
| 1437442_at | *Pcdh7* | Protocadherin 7 | -1.52 | 0.000139 |
| 1453571_at | *Depdc6* | DEP domain containing 6 | -1.51 | 0.000325 |
| 1435828_at | *Maf* | avian musculoaponeurotic fibrosarcoma (v-maf) AS42 oncogene homolog | -1.51 | 0.003319 |
| 1450782_at | *Wnt4* | wingless-related MMTV integration site 4 | -1.51 | 0.000683 |
| 1437401_at | *Igf1* | insulin-like growth factor 1 | -1.51 | 0.000553 |

** P* value of t-test using Bayesian standard deviations in Cyber-T.
