# Supplementary material for: Transcriptomic responses in mouse brain exposed to chronic excess of the neurotransmitter glutamate
Source: BMC Genomics. 2010 Jun 7;11:360. doi: 10.1186/1471-2164-11-360 (PMC2896956; doi:10.1186/1471-2164-11-360)
Supplement: Additional file 3 — IPA bio-functions and genes in Glud1 Tg. Complete list of statistically significant bio-functions and associated genes from the IPA analysis. The bio-functions are reported in Figure 1D. [file 1471-2164-11-360-S3.DOC]

**Additional Table 3 – Genes associated with the over-represented IPA bio-functions reported in Fig. 1D**

| **Canonical Functions** | **Associated Genes** |
| --- | --- |
| **Cell Signaling**  Up-regulated genes (Total Number: 61)  Down-regulated genes (23)  **Molecular Transport**  Up-regulated genes (41)  Down-regulated genes (18)  **Cellular Assembly and Organization**  Up-regulated genes (100)  Down-regulated genes (26)  **Nervous System Development and Function**  Up-regulated genes (103)  Down-regulated genes (28)  **Amino Acid Metabolism**  Up-regulated genes (35)  Down-regulated genes (6)  **Cell Death**  Up-regulated genes (99)  Down-regulated genes (37)  **Behavior**  Up-regulated genes (30)  **RNA Post-Transcriptional Modification**  Up-regulated genes (20)  **Organismal Survival**  Up-regulated genes (44)  **RNA Trafficking**  Up-regulated genes (3)  **DNA Replication, Recombination, and Repair**  Up-regulated genes (13) | *Actb, Acvr2a, Akt3, Apc, Arf3, Arpc2, Atp2b2, Atp2c1, Cacna1c, Cacna1d, Cacna1h, Cacna2d1, Cacnb2, Camk2a, Camk2b, Capn10, Capn3, Dlg4, Dusp6, Fut8, Gabbr1, Gja1, Gnaq, Gng2, Grin2a, Grin2b, Hipk2, Il1rap, Iqgap2, Itga8, Kpnb1, Map2k4, Mapk14, Mapk8, Mbp, Ncam1, Ndst1, Nfs1, Ntrk2, Ntrk3, Picalm, Plcl1, Ppp3ca, Prkca, Ptk2, Ptk2b, Ptprd, Ptprj, Rasgrp1, Rgs14, Rims1, Rraga, Ryr1, Spred2, Tnfrsf25, Trpc4, Trpm7, Tspan5, Wasf3, Wasl, Ywhae*  *Adra2a, Cacnb4, Calca, Camk2d, Cast, Cav2, Cckbr, Cdc42ep1, Cdh13, Dcn, Gap43, Igf1, Nts, Reln, Rfxank, Rgs17, Rgs2, Sparc, Timp2, Tnnt2, Trhr, Vip, Wnt7a*  *Anp32a, Atp1a1, Atp2b2, Atp2c1, Atxn1, Cacna1c, Cacna1d, Cacna1h, Cacna2d1, Cacnb2, Camk2a, Camk2b, Erc2, Frap1, Gja1, Gnaq, Gpam, Grin2a, Grin2b, Htr1a, Kcna6, Kcnd2, Kcnip2, Kcnq1, Kcnq2, Lyk5, Nrxn3, Ntrk2, Ppp3ca, Ptk2, Rgs14, Ryr1, Scd, Scn2a, Scn8a, Smg6, Snca, Trpc4, Trpm7, Uhmk1, Ywhae*  *Adra2a, Atp1a2, Calca, Camk2d, Cast, Cav2, Cckbr, Dcn, Gap43, Igf1, Igfbp3, Nts, Rgs2, Satb1, Slc17a6, Trhr, Vip, Wnt7a*  *Actb, Adam17, Add1, Ank3, Anp32a, Ap2a2, Apc, Arf3, Arhgef12, Arhgef2, Arpc2, Atp2b2, Atp2c1, Bcl11b, Bin1, Cacna1c, Cacnb2, Camk2a, Cap2, Capn10, Centb2, Centg2, Clasp1, Clip1, Coro1c, Cttn, Diaph1, Dlg1, Dlg4, Dmd, Dnaja3, Dpysl2, Dsp, Dst, Eif4a1, Eif4e, Enah, Enc1, Epha4, Erc2, Exoc5, Frap1, Gas7, Gja1, Gnaq, Gng2, Gosr1, Gpm6a, Grin2a, Grip1, Htr1a, Itga8, Junb, Kalrn, Kcnq1, Klf2, Kpnb1, Ksr1, Map2, Map7, Mapk14, Mapk8, Mbp, Myo9b, Napg, Nav1, Ncam1, Ndel1, Neo1, Net1, Nfia, Nisch, Nmt1, Nrcam, Nrp1, Ntrk2, Ntrk3, Pafah1b1, Pex5l, Picalm, Prkg1, Psap, Ptk2, Ptk2b, Reps1, Rictor, Rims1, Rps6kb1, Rtn4, Sec24b, Sema5a, Slit3, Snca, Spire1, Top2b, Tpm1, Trpc4, Wasf3, Wasl, Ywhaz*  *Cacnb4, Calca, Cast, Cav2, Cd24, Cd44, Cdc42ep1, Cdh13, Cit, Cntn4, Ctgf, Dcx, Efna5, Gap43, Grin3a, Igf1, Igfbp3, Klf5, Nts, Ppard, Reln, Rgs2, Sdc2, Sparc, Stmn1, Wnt7a*  *Actb, Adam17, Akt3, Ank3, Anp32a, Apc, Aplp2, Arc, Atf2, Atp2b2, Atrn, Atrx, B3gnt2, Bcl11b, Bin1, Cacna1c, Cacna1h, Cacnb2, Calb1, Camk2a, Diaph1, Dlg2, Dlg4, Dmd, Dnaja3, Dpysl2, Dtna, Dyrk1a, Egr1, Egr2, Enah, Enc1, Epha4, Epha7, Erc2, Fos, Frap1, Gabbr1, Gabrb2, Gabrb3, Gas7, Gja1, Gmfb, Gnaq, Gpm6a, Grin2a, Grin2b, Grip1, Htr1a, Ifrd1, Itga8, Junb, Kalrn, Kcnd2, Kcnq2, Kif5c, Ksr1, Map2, Map2k4, Mapk14, Mapk8, Mbp, Nav1, Ncam1, Ndel1, Ndst1, Nfia, Nfib, Nptx1, Nrcam, Nrp1, Nrxn3, Ntrk2, Ntrk3, Oprs1, Pafah1b1, Pcdhb13, Per2, Picalm, Polb, Ppp3ca, Prkca, Prkg1, Psap, Ptk2, Ptk2b, Ptprd, Rims1, Rtn4, Scn2a, Scn8a, Sema5a, Ski, Slit3, Snca, Sptbn1, Stx1b, Top2b, Ube3a, Ywhae, Ywhag, Zbtb16, Zeb2*  *Arx, C5orf13, Calca, Cbln1, Cd24, Cit, Cntn4, Cux1, Dcx, Efna5, Fgf12, Gap43, Grin3a, Hes5, Hrh1, Igf1, Lhx2, Mef2c, Ninj1, Nr2f2, Pbx3, Pex13, Reln, Stmn1, Timp2, Unc13c, Vip, Wnt7a*  *Acvr1, Acvr2a, Akt3, B3gnt2, Brsk2, Btg2, Camk2a, Camk2b, Col4a3bp, Csnk1a1, Dusp6, Dyrk1a, Fer, Frap1, Gmfb, Hipk2, Kalrn, Large, Lyk5, Mapk14, Mapk8, Mtmr3, Ndst1, Phka2, Ppp3ca, Prkca, Prkg1, Ptk2b, Ptprd, Ptprj, Rps6kb1, Srpk2, St6galnac3, Tlk2, Trpm7*  *Ass1, Igf1, Igfbp3, Nts, Reln, Slc17a6*  *Acvr1, Akt3, Aldh2, Ap2a2, Apc, Arc, Atf2, Atf6, Atp1a1, Atp2c1, Atrx, Atxn1, Bclaf1, Bin1, Btg2, Cacna1c, Cacnb2, Calb1, Camk2a, Capn10, Chka, Csnk1a1, Dmd, Dnaja3, Dsp, Dusp6, Egr1, Egr2, Eif4e, Fem1b, Fer, Fos, Frap1, Fubp1, Fus, Gabbr1, Gja1, Gmfb, Gnaq, Gng2, Grin2a, Hdac2, Hipk2, Hpca, Junb, Klf2, Ksr1, Lrig1, Map2k4, Mapk14, Mapk8, Mbp, Mef2a, Mgat3, Ncam1, Ndel1, Ndst1, Nptx1, Nr4a1, Nrcam, Nrf1, Nrp1, Ntrk2, Ntrk3, Pafah1b1, Phip, Polb, Ppp1r13b, Ppp3ca, Prkca, Psap, Ptk2, Ptk2b, Rad23b, Rasgrp1, Rbm5, Rps6kb1, Rraga, Rtn4, Scn2a, Scn3b, Sh3rf1, Ski, Slk, Snca, Tacc1, Tfrc, Tnfrsf25, Tnks2, Tpm1, Ube2k, Ubtf, Usp7, Vps33a, Ywhae, Yy1, Zbtb16, Zeb2, Zfr*  *Acvr2b, Angpt1, Anxa1, Atp1a2, Calca, Camk2d, Casp1, Cast, Cd24, Cd44, Cit, Ctgf, Dcn, Ecop, Etv6, Gulp1, Hoxc6, Igf1, Igfbp3, Igfbp6, Klf5, Mef2c, Nedd9, Phlda1, Plagl1, Ppard, S100a10, Satb1, Sdc2, Sparc, Stmn1, Tgm2, Timp2, Tmsb10, Vip, Wnt7a, Wwox*  *Aplp2, Arc, Atp2b2, Atxn1, Cacna1c, Calb1, Camk2a, Dlg2, Dlg4, Dyrk1a, Egr1, Egr2, Epha4, Gabbr1, Gabrb3, Gnaq, Grin2a, Grin2b, Htr1a, Itga8, Junb, Ntrk2, Oprs1, Pafah1b1, Plcl1, Prkg1, Ptk2, Ptprd, Scn8a, Snca*  *Adar, Cdc40, Cpsf6, Cugbp2, Eif4a1, Eif4e, Hnrnpr, Hnrpab, Rbm3, Rbm5, Rps6kb1, Sfpq, Sfrs10, Sfrs11, Sfrs12, Sfrs8, Snrp70, Srpk2, Syncrip, Tra2a*  *Adar, Akt3, Apc, Aplp2, Cacnb2, Dmd, Dnaja3, Dsp, Dyrk1a, Egr2, Enah, Fus, Fut8, Gabrb3, Gja1, Gnaq, Grip1, Hapln1, Hipk2, Klf2, Magi2, Map2k4, Mapk14, Mapk8, Mef2a, Nfia, Nrf1, Nrp1, Ntrk2, Ntrk3, Pbx1, Pnpla6, Polb, Prkca, Ptk2, Ptprs, Rad23b, Ski, Spred2, Top2b, Wasl, Wdr1, Yy1, Zfr*  *Eif3a, Eif4a1, Eif4e*  *Atf2, Atp1a1, Fos, Gnaq, Gng2, Iqgap2, Kpnb1, Mapk8, Myo9b, Ptk2, Ptk2b, Ubtf, Yy1* |
