# Supplementary material for: Transcriptomic responses in mouse brain exposed to chronic excess of the neurotransmitter glutamate
Source: BMC Genomics. 2010 Jun 7;11:360. doi: 10.1186/1471-2164-11-360 (PMC2896956; doi:10.1186/1471-2164-11-360)
Supplement: Additional file 4 — IPA pathways and genes in Glud1 Tg. Complete list of statistically significant canonical pathways and associated genes from the IPA analysis. The canonical pathways are reported in Figure 1E. [file 1471-2164-11-360-S4.DOC]

**Additional Table 4 – Genes associated with the over-represented IPA canonical pathways reported in Fig. 1E**

| **Canonical Pathways** | **Associated Genes** |
| --- | --- |
| **Huntington's Disease Signaling**  Up-regulated genes (Total number: 17)  Down-regulated genes (5)  **Axonal Guidance Signaling**  Up-regulated genes (23)  Down-regulated genes (10)  **Calcium Signaling**  Up-regulated genes (14)  Down-regulated genes (4)  **Chemokine Signaling**  Up-regulated genes (9)  Down-regulated genes (1)  **Ephrin Receptor Signaling**  Up-regulated genes (14)  Down-regulated genes (6)  **Synaptic Long Term Potentiation**  Up-regulated genes (9)  Down-regulated genes (2)  **Integrin Signaling**  Up-regulated genes (12)  **NRF2-mediated Oxidative Stress Response**  Up-regulated genes (11)  Down-regulated genes (3)  **Neuregulin Signaling**  Up-regulated genes (7)  Down-regulated genes (1)  **Neurotrophin/TRK Signaling**  Up-regulated genes (6)  Down-regulated genes (1)  **Toll-like Receptor Signaling**  Up-regulated genes (5) | *Akt3, Ap2a2, Atf2, Capn10, Capn3, Dlg4, Frap1, Gnaq, Gng2, Gosr1, Grin2b, Hdac2, Map2k4, Mapk8, Napg, Prkca, Snca*  *Casp1, Gng4, Igf1, Pik3r5, Tgm2*  *Adam17, Akt3, Arhgef12, Arpc2, Dpysl2, Eif4e, Epha3, Epha4, Epha6, Epha7, Gnaq, Gng2, Kalrn, Nrp1, Ntrk2, Ntrk3, Ppp3ca, Prkca, Ptk2, Rtn4, Sema5a, Slit3, Wasl*  *Arhgef15, Efna5, Gng4, Igf1, Pik3r5, Sdc2, Sema4a, Unc5d, Wnt4, Wnt7a*  *Atf2, Atp2b2, Atp2c1, Camk2a, Camk2b, Grin2a, Grin2b, Hdac2, Mef2a, Ppp3ca, Ryr1, Slc8a2, Tpm1, Trpc4*  *Camk2d, Grin3a, Mef2c, Tnnt2*  *Camk2a, Camk2b, Fos, Gnaq, Mapk14, Mapk8, Prkca, Ptk2, Ptk2b*  *Camk2d*  *Akt3, Arpc2, Atf2, Epha3, Epha4, Epha6, Epha7, Gnaq, Gng2, Grin2a, Grin2b, Kalrn, Ptk2, Wasl*  *Angpt1, Arhgef15, Efna5, Gng4, Sdc2, Sh2d3c*  *Atf2, Cacna1c, Camk2a, Camk2b, Gnaq, Grin2a, Grin2b, Ppp3ca, Prkca*  *Camk2d, Grin3a*  *Actb, Akt3, Arf3, Arpc2, Capn10, Capn3, Itga8, Map2k4, Mapk8, Ptk2, Tspan5, Wasl*  *Actb, Dnaja3, Dnajb5, Dnajc1, Fos, Junb, Map2k4, Mapk14, Mapk8, Prkca, Ube2k*  *Maf, Pik3r5, Slc35a2*  *Adam17, Akt3, Dlg4, Frap1, Nrg3, Prkca, Rps6kb1*  *Dcn*  *Atf2, Fos, Map2k4, Mapk8, Ntrk2, Ntrk3*  *Pik3r5*  *Fos, Map2k4, Mapk14, Mapk8, Tollip* |
